# Capacity of soil bacteria to reach the phyllosphere and convergence of floral communities despite soil microbiota variation

**DOI:** 10.1073/pnas.2100150118

**Published:** 2021-10-07

**Authors:** Julien Massoni, Miriam Bortfeld-Miller, Alex Widmer, Julia A. Vorholt

**Affiliations:** ^a^Institute of Microbiology, Department of Biology, ETH Zurich, 8093 Zurich, Switzerland;; ^b^Center for Adaptation to a Changing Environment, ETH Zurich, 8092 Zurich, Switzerland;; ^c^Institute of Integrative Biology, ETH Zurich, 8092 Zurich, Switzerland

**Keywords:** phyllosphere, bacterial communities, soil, flower

## Abstract

Leaves and flowers are colonized by diverse bacteria that impact plant fitness and evolution. Although the structure of these microbial communities is becoming well-characterized, various aspects of their environmental origin and selection by plants remain uncertain, such as the relative proportion of soilborne bacteria in phyllosphere communities. Here, to address this issue and to provide experimental support for bacteria being filtered by flowers, we conducted common-garden experiments outside and under gnotobiotic conditions. We grew *Arabidopsis thaliana* in a soil substitute and added two microbial communities from natural soils. We estimated that at least 25% of the phyllosphere bacteria collected from the plants grown in the open environment were also detected in the controlled conditions, in which bacteria could reach leaves and flowers only from the soil. These taxa represented more than 40% of the communities based on amplicon sequencing. Unsupervised hierarchical clustering approaches supported the convergence of all floral microbiota, and 24 of the 28 bacteria responsible for this pattern belonged to the Burkholderiaceae family, which includes known plant pathogens and plant growth-promoting members. We anticipate that our study will foster future investigations regarding the routes used by soil microbes to reach leaves and flowers, the ubiquity of the environmental filtering of Burkholderiaceae across plant species and environments, and the potential functional effects of the accumulation of these bacteria in the reproductive organs of flowering plants.

## Significance

The role of flowers as environmental filters for bacterial communities and the provenance of bacteria in the phyllosphere are currently poorly understood. We experimentally tested the effect of induced variation in soil communities on the microbiota of plant organs. We identified soil-derived bacteria in the phyllosphere and show a strong convergence of floral communities with an enrichment of members of the Burkholderiaceae family. This finding highlights a potential role of the flower in shaping the interaction between plants and a bacterial family known to harbor both plant pathogens and growth-promoting strains. Because the flower involves host–symbiont feedback, the selection of specific bacteria by the reproductive organs of angiosperms could be relevant for the modulation of fruit and seed production.

All plants are colonized by diverse prokaryotic and eukaryotic microorganisms. While root microbiota affect plant development, leaf and floral microorganisms can also provide services, such as protection against pathogens or interference with plant–insect interactions (1–4). These functional roles illustrate the importance of these microorganisms for the development and evolution of their hosts. While the diversity of bacteria and fungi colonizing leaves and flowers has been extensively explored (5–10), the environmental sources of these microorganisms and their relative contributions are still controversial (11, 12). Insights into the origin of phyllosphere bacteria will contribute to the understanding of the life cycles, ecology, and adaptation of plants and their microbiota. Various environmental sources have been suggested, and seed banks including soil are often hypothesized to be the major sources of bacteria detected in the phyllosphere (13–16). This assumption is supported by the strong resemblance between soil and leaf microbiota at the beginning of the growing season (14). However, this similarity was also observed when phyllosphere microbiota were compared with aerial communities (17). The overlap in composition between communities of the phyllosphere, soil, and roots has been used as a further argument for the large contribution by soil communities (5, 16). This last interpretation does not consider potential concurrent colonization of soil and aerial parts of plants or additional sources. Regarding floral communities, insects (e.g., pollinators) have also been suggested as important vectors for bacterial taxa, but their contribution does not suffice to explain the bacterial diversity observed in floral communities (11, 18–20). In contrast to comparative studies with naturally grown plants, experimental approaches that address the origin of the phyllosphere microbiota are still rare. A recent experimental study involved the transplantation of adult plants from one soil to another, and the resulting shifts in leaf communities suggest continued colonization of leaves by soil bacteria (13). Despite this previous work, the contribution of leaf and floral bacteria, which have the ability to migrate from the soil, is still uncertain. The influence of soil microbiota variation alone, independent of other edaphic conditions, on phyllosphere communities also remains to be addressed.

Another important aspect in our understanding of plant microbiota concerns the observed differences between the bacterial communities of roots, leaves, and flowers, not only among each other but also with respect to the surrounding microbial context (5, 6, 14, 16, 17, 21–23). The observed variations have been interpreted as being due to environmental filtering exerted by leaves and flowers on the diversity of bacteria present in the environment. However, the identity of the bacteria being filtered still needs to be investigated experimentally.

**Fig. 1.**
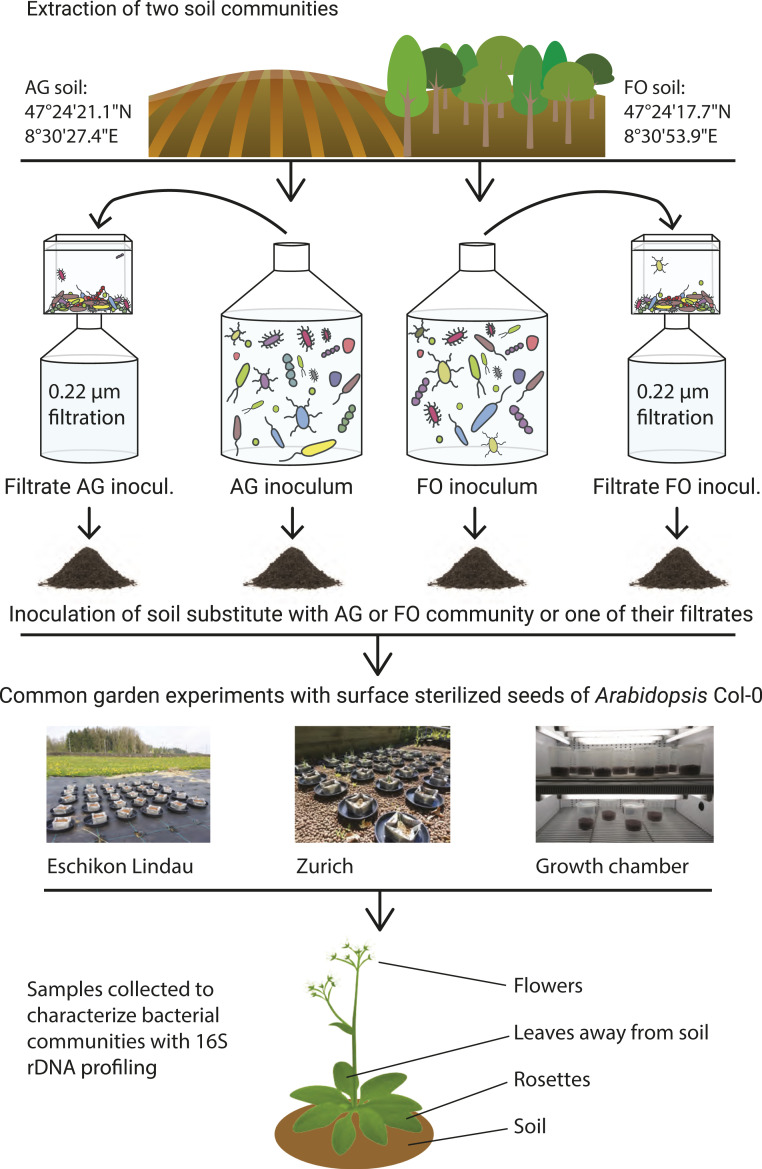
Experimental design.

In the present study, we conducted an experiment to address the origin of microorganisms in the phyllosphere. The underlying rationale was that if the phyllosphere organs filter for specific bacterial groups, differences in surrounding microbial communities should not prevent the convergence of the communities. The experimental design was such that it also allowed us to evaluate the proportion of phyllosphere bacteria that can reach leaves and flowers from the soil. We asked the following questions. 1) What is the proportion of bacterial taxa detected on leaves and flowers that have the ability to reach the phyllosphere from soil? 2) Does variation in the soil microbiota alone drive bacterial community divergence in leaves? 3) Does variation in the soil microbiota alone drive bacterial community divergence in flowers? 4) Do specific organ communities converge despite microbial variations in the microbial context surrounding the plant? 5) Which taxa support these potential convergent organ communities? We conducted common-garden experiments in both gnotobiotic and open conditions (Fig. 1). In each experiment, the soil microbial communities were the only varying parameter. Soil, leaf, and floral communities were characterized by 16S ribosomal RNA (rRNA) gene profiling using 100% identity to define taxonomic units, also termed amplicon sequence variants (ASVs) (24). Unsupervised hierarchical clustering approaches coupled with bootstrap and serial rarefaction sensitivity tests were used to measure the impact of soil communities on the bacterial microbiota of leaves and flowers; in addition, the taxa underlying the convergence of floral communities were identified. We discuss our results in the context of microbial migration, environmental selection, and the ecology of Burkholderiaceae being filtered by the floral environment.

## Results

### Proportion of Phyllosphere Bacteria that Have the Potential to Colonize from Soil.

To measure the influence of the soil microbiota on bacterial communities in the phyllosphere, we conducted common-garden experiments using *Arabidopsis thaliana* Col-0 and an experimental treatment consisting of growing these plants in different microbial communities of soil (Fig. 1). The experiments were replicated at two locations outside and in a growth chamber (*Methods*). At the outside locations, the microbial communities detected in the plants could have come from any environmental source (e.g., soil, air, rain, or insects), whereas the microboxes (*Methods*) used in the growth chamber prevented migration of microorganisms toward the phyllosphere from any source but the soil. We used a standard axenic soil substitute inoculated with either a microbial community extracted from agricultural field soil (AG) or a community extracted from forest soil (FO) (*Methods*). The soil substitute allowed us to fix the soil characteristics with the exception of the soil microbiota. To control for the potential impact of wild-soil residual chemistry extracted together with the bacterial cells, we filtered the suspensions through a 0.22-µm filter and used the filtrates in additional soil treatments (*Methods* and Fig. 1). At each common-garden location, we had five replicates for each treatment type. Plants grew in all the pots and microboxes at the Zurich location and in the growth chamber; however, virtually no plants germinated at the Eschikon Lindau location. This is either due to the harsher environmental conditions at this location or seeds being blown away due to windy conditions. When the plants reached the adult stage, we took soil samples and collected rosettes of leaves as well as inner leaves (which were not in contact with the soil) from the growth chamber. The adult plant specimens at the outside location were too small to collect the inner leaves not in contact with the soil. During the blooming stage, at the outside location and in the growth chamber, we collected soil samples, rosettes (i.e., all leaves), inflorescences, and leaves not in contact with the soil; 16S rDNA amplicon sequencing was used to detect endophytic and epiphytic bacteria in all the samples. After raw data treatment (*Methods*) and the removal of controls used to build the DNA library, our data included 374 samples and 4,063,848 reads, with an average of 10,865 reads per sample. The 182 samples collected at the outside location had more reads on average than the 165 samples collected in the growth chamber (12,550 versus 7,869, respectively).

We detected 6,010 different ASVs at a 100% identity threshold, with 5,617 of these taxa being detected at the outside location and 5,239 detected in the growth chamber. An estimation of the possible contamination from seeds despite sterilization revealed that this fraction was small, with four ASVs potentially originating from this contamination source (see *Methods* and *SI Appendix*, *Text* for a detailed assessment of the control samples).

**Fig. 2.**
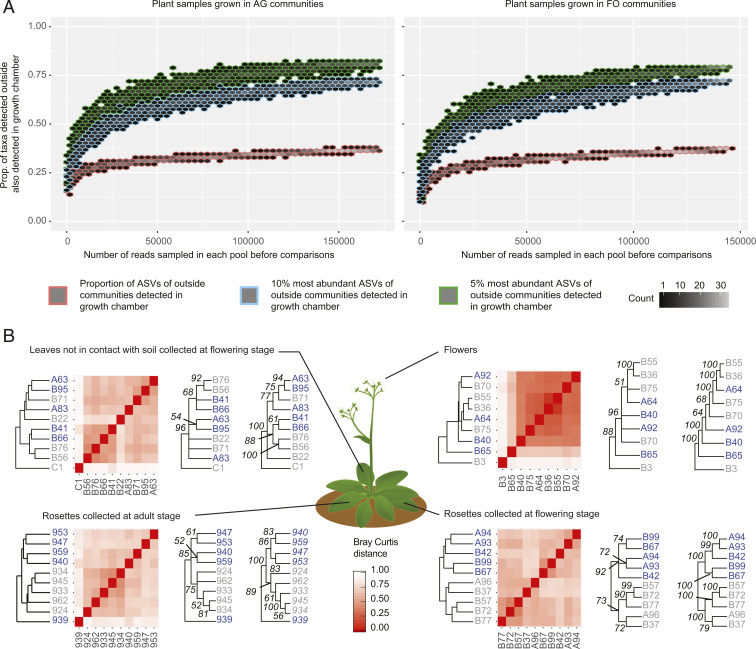
Soil community influence on the phyllosphere. (*A*) Hexagonal heatmap of the proportion of ASVs detected in the phyllosphere at the outside location also detected in phyllosphere communities in the growth chamber. All samples collected outside were combined in one pool, and those collected inside were combined in another before comparisons. (*B*) Unsupervised hierarchical clustering of phyllosphere communities (identified by their sample number) collected at outside locations, using the Bray–Curtis metric as a measure of dissimilarity. The data were all rarefied according to the number of reads from the smallest plant sample collected outside. Each panel consists of a dendrogram and a heatmap generated from rarefied data and two majority-rule consensuses: the first one summarizes the hierarchical clustering analyses based on 1,000 bootstrapped datasets, and the second one summarizes the hierarchical clustering analyses based on 1,000 rarefied datasets. The nodes are labeled with the percentage of recovery of each cluster across these iterative analyses. The tips are colored according to the community used to inoculate the soil in which the plants were grown (gray, AG community; blue, FO community). prop., proportion.

To estimate the proportion of bacteria with the potential to reach leaves and flowers from the soil, we identified the bacteria that were detected in the plant samples collected outside and in the growth chamber. Because the microboxes used in the growth chamber allow bacterial transfer from soil only, the taxa detected on leaves and flowers in this environment have the capacity to migrate actively or passively from the soil to the phyllosphere. To define the overlap between the outside and inside communities, we combined all samples collected at one location in one pool and those from the other site in another. We conducted comparison series after randomly sampling the same number of reads in each pool using an incremental 100-read procedure. The results of these comparisons converged, showing that 37% of the bacteria detected in the phyllosphere in the outside location were also detected in the phyllosphere in the growth chamber (this value was identical for the plants grown in soils inoculated with the AG and FO communities; Fig. 2*A*). When we used the same procedure to examine the communities of each organ separately, the results converged on 25% of the bacteria detected outside and in the growth chamber (*SI Appendix*, Fig. S1). When we restricted this analysis to the most abundant taxa in the communities (i.e., those in the top 10 and 5%, representing more than 75 and 85% of the total number of reads in the communities, respectively), more than 75% of the bacteria detected outside were also detected inside for the AG and FO communities (Fig. 2*A*).

To account for spurious presence generated by potential sample cross-contaminations and index-switching events, we repeated the comparison procedure with a presence threshold of three reads in nonrarefied data (i.e., all relative abundances of fewer than three reads were set as zeros). We defined this threshold from an exploration of the Zymo mock community to estimate that ASVs not expected to be part of this community had an average of two reads (*Methods*). From this transformed dataset, the results of comparisons converged to about 30% of the bacteria detected in the plants collected outside being also detected in the plants grown in the growth chamber (31 and 27% for AG and FO communities, respectively; *SI Appendix*, Fig. S2). When we restricted this analysis to the most abundant taxa in the communities (i.e., those in the top 10%), more than 59 and 53% of the bacteria detected outside were also detected inside for the AG and FO communities, respectively (*SI Appendix*, Fig. S2). In bacterial communities found on adult rosettes, an average of 51 and 40% of the reads belonged to soil ASVs that can reach these organs grown in AG and FO communities, respectively (*SI Appendix*, Fig. S3). At the flowering stage, these proportions were 54 and 61% in AG and FO rosettes, respectively. The soil ASVs able to reach the leaves away from soil represented 60 and 41% of the total number of reads of AG and FO leaf communities, respectively (*SI Appendix*, Fig. S3). Finally, soil bacteria able to reach flowers dominated floral communities in plants grown in AG and FO communities (69 and 73% of reads, respectively; *SI Appendix*, Fig. S3).

### Impact of Soil Microbiota Variation on Bacterial Community Divergence in the Phyllosphere.

**Fig. 3.**
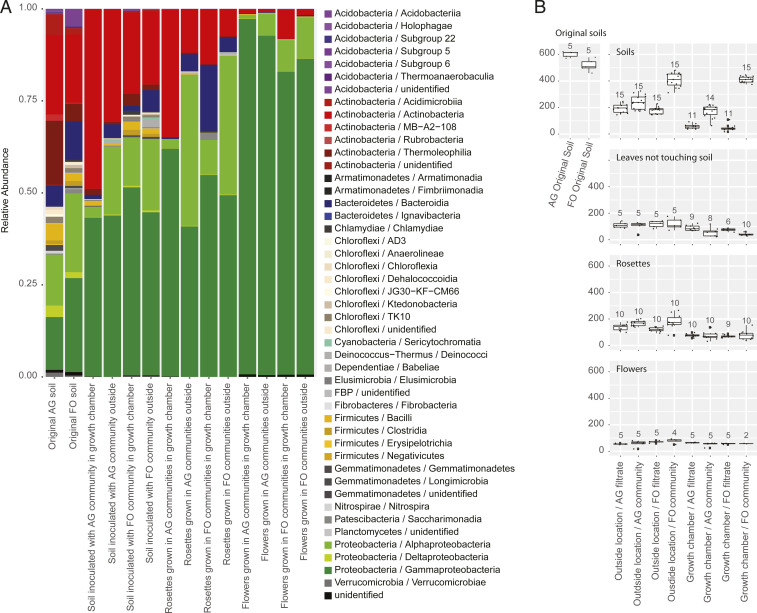
Bacterial diversity. (*A*) Relative abundances of bacterial lineages in the different types of samples. (*B*) Community richness in the different types of samples and different location and soil treatment combinations (data were rarefied; numbers of samples are indicated above each boxplot; centerline, median; box limits, upper and lower quartiles; whiskers, 1.5× interquartile range; points, outliers).

The original soils and inoculated soil substitutes differed in their community composition and structure, as we showed by unsupervised clustering analysis with the Bray–Curtis distance metric after rarefaction according to the depth of the smallest sample (1,016 reads) (Fig. 3 and *SI Appendix*, Fig. S4). To provide statistical support for the clusters, we bootstrapped this rarefied dataset 1,000 times and repeated the cluster analyses with each resulting dataset. This approach allowed us to provide the frequency of recovery of each cluster across these 1,000 analyses. Because rarefaction also involves random sampling of sequences that could lead to clusters supported by chance, we also conducted 1,000 random rarefaction and cluster analyses and calculated the frequencies of recovery of each cluster across these analyses. The communities in the original soils clustered according to their origin, with high statistical support (100% of bootstrap and sequential rarefaction analyses; *SI Appendix*, Fig. S4). The communities in the soil inoculated with the AG community formed two well-supported clusters. One included all the communities from the growth chamber (100% of bootstrap and sequential rarefaction analyses; cluster B in *SI Appendix*, Fig. S4), and one included all the communities from the outside location (86 and 100% of bootstrap and sequential rarefaction analyses, respectively; cluster D in *SI Appendix*, Fig. S4). The communities in the soil inoculated with the FO microbiota also formed two well-supported clusters. One included all the communities collected in the growth chamber (100% of bootstrap and sequential rarefaction analyses; cluster E in *SI Appendix*, Fig. S4), and one included all the communities collected at the outside location (93 and 100% of bootstrap and serial rarefaction analyses, respectively; cluster F in *SI Appendix*, Fig. S4). In all these analyses, the soil communities did not cluster according to the time of collection (i.e., when plants were adults or blooming; *SI Appendix*, Fig. S4).

Most likely due to the release of environmental DNA and potentially because cells were not all excluded by the filtration procedure, the soil inoculated with the filtrates of the AG and FO inocula was not free of DNA. However, the communities in the soils inoculated with the filtrates of the wild communities and collected in the growth chamber did not cluster consistently together and had unstable positions across the bootstrapped and sequential rarefaction analyses (clusters labeled A in *SI Appendix*, Fig. S4). This result suggests the efficient removal of most living bacterial cells from these filtrates. This is further supported by the finding that all the communities in the soils inoculated with the filtrates of AG and FO microbiota and collected from the outside location clustered together in a well-supported group and were not separated according to the filtered inoculum (82 and 100% of bootstrapping and sequential rarefaction, respectively; cluster C in *SI Appendix*, Fig. S4).

Regarding the microbiota composition, the original soils collected in the agricultural field and forest were dominated by Actinobacteria and Proteobacteria, which together represented more than 70% of the total number of sequence reads in the communities, while Acidobacteria, Actinobacteria, Bacteroidetes, Firmicutes, and Proteobacteria represented more than 90% of the total reads in both original soils (91 and 92% in the AG and FO communities, respectively; Fig. 3*A*). These bacterial taxa also dominated in all the soil substitutes inoculated with the communities extracted from these natural soils (Fig. 3*A*). The plant communities were also dominated by the same bacterial taxa, with a higher relative abundance of Proteobacteria than in the soil samples. This is especially the case for the floral samples, in which Proteobacteria represented between 91 and 98% of the total communities (Fig. 3*A*). With regard to richness, the soil housed more taxa than the leaves and flowers (Fig. 3*B*).

To measure whether soil microbiota variations led to detectable divergences in the outside AG and FO phyllosphere communities, we performed unsupervised clustering analyses of the communities on each plant organ separately (rosettes, leaves not in contact with the soil, and flowers). If the organ microbiota diverged according to the soil microbiota, we would expect the communities of plants grown in one soil type to cluster together, separate from those grown in the other soil types. To compare the results obtained with the different organs, we rarefied all the samples at 1,084 reads (the size of the smallest plant sample collected outside). Only rosettes clustered according to the soil communities in which they grew at the adult stage (75 and 89% of the bootstrap and sequential rarefaction analyses, respectively; Fig. 2*B*) and at the flowering stage (more than 70% of the bootstrap analyses and 100% of iterative rarefaction analyses; Fig. 2*B*). When we conducted the same analyses with rosettes from plants grown in soil inoculated with filtrates of AG and FO communities, the samples were not classified according to the filtrate used. This latter result suggests the absence of an influence of the residual chemistry extracted from the wild soil samples and confirms that we are indeed measuring the influence of the microbial communities (*SI Appendix*, Fig. S5). Increasing the number of reads to the size of the smallest sample in each organ and developmental stage combination led to results consistent with the 1,084 rarefied samples (*SI Appendix*, Fig. S6).

### Convergence of Floral Communities Despite Environmental–Microbial Variations.

**Fig. 4.**
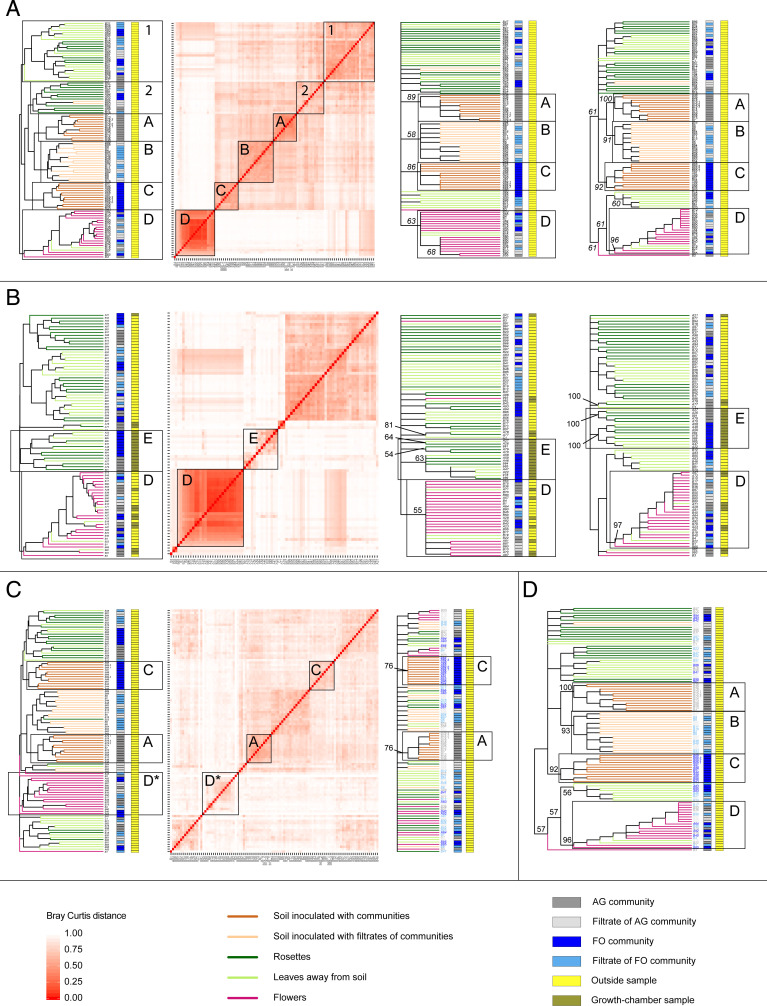
Convergence of phyllosphere communities. Unsupervised hierarchical clustering analyses of bacterial communities. The communities are identified by their sample number. (*A*) Communities of all the samples collected outside at the flowering stage. The first dendrogram and heatmap were generated from one rarefied dataset; the second and third dendrograms are majority-rule consensuses of hierarchical clustering analyses conducted with 1,000 bootstrapped and 1,000 rarefied datasets, respectively. (*B*) Communities of the plant samples collected during the flowering stage at outside locations plus communities of the plant samples grown in the growth chamber in soil communities. The layout is the same as that in *A*. (*C*) Communities of all the samples collected outside during the flowering stage after the removal of the candidate ASVs responsible for the convergence of the floral cluster. The first dendrogram and heatmap were generated with a rarefied dataset, and the majority-rule consensus was obtained from hierarchical clustering analyses of 1,000 rarefied datasets. (*D*) Majority-rule consensus of hierarchical clustering analyses after 1,000 subsequent removals of 28 ASVs (not the ASV candidates responsible for the clustering of floral communities observed in *A*). The numbers presented at the nodes of the majority-rule consensus dendrograms are the percentage of clusters recovered across analyses conducted with 1,000 bootstrapped datasets and 1,000 rarefied datasets. The numbered clusters (clusters 1 and 2) are not supported with bootstrapped and 1,000 rarefied datasets. The clusters supported with bootstrap and 1,000 rarefied datasets are labeled as described in the text to allow comparisons across panels of the figure. Asterisks indicate that the relative positions of tips give the misleading impression that cluster D exists in the first dendrogram of *C*.

To estimate whether the microbial communities on the rosettes, leaves not in contact with the soil, or flowers converged according to organ type, we included all the soil and blooming plant samples collected outside in one unsupervised hierarchical clustering analysis. We chose this classification method because it does not involve a priori assumptions in the definition and number of groups in which samples are assigned. The three soil clusters identified above were recovered in this new analysis, leaf communities did not form any cluster, and floral communities grouped together (cluster D in Fig. 4*A*). In other words, the flower communities were more similar to each other than to any other plant or soil community. This floral cluster included all the floral communities and two communities from leaves that did not contact the soil. Within this floral cluster, the samples did not form subclusters according to the soil communities in which they grew. Except for one floral community (sample B3), both the bootstrap and sequential rarefaction analyses provided good statistical support for the clustering of the floral communities (63 and 96% of analyses, respectively; Fig. 4*A*). Interestingly, sample B3 was statistically supported as part of the floral cluster when we used a binary Bray–Curtis metric, which only considers the presence or absence of taxa (*SI Appendix*, Fig. S7). We repeated this analysis with the Bray–Curtis distance metric, all the blooming plant samples collected outside, and all the blooming plant samples collected in the growth chamber and grown in the AG and FO communities. With the exception of one, all the communities from flowers grown in the growth chamber were nested in the cluster of floral communities collected outside (55 and 97% of bootstrap and sequential rarefaction analyses, respectively; cluster D in Fig. 4*B*). This result shows that the floral communities of the plants in the growth chamber are more similar to the floral communities of the plants at the outside location than to any other plant communities collected at either location. In contrast, the leaf communities from the plants grown in the growth chamber clustered separate from those from the plants grown in the outside location and were not statistically supported as one cluster (cluster E in Fig. 4*B*).

### Bacterial Taxa Responsible for the Convergence of Floral Communities.

Because the plants in the growth chamber were not exposed to natural biotic and abiotic conditions (e.g., air microbiota, temperature variation, radiation, and precipitation), the relative abundance of some of their bacterial taxa might not reflect natural conditions. For this reason, we identified the bacterial taxa in the communities from the outside samples that supported the floral community cluster. We generated 1,000 datasets rarefied at 1,084 reads. We conducted independent unsupervised hierarchical clustering analyses across these 1,000 datasets but with the data of each taxon separate rather than the entire communities as above. In total, 28 taxa were identified as being responsible for the convergence of the floral communities, as their distribution of relative abundances across the samples supported the clustering of floral communities in all 1,000 rarefied datasets. This conservative approach, which consisted of excluding any bacteria not supporting floral clustering in all the rarefied datasets, allowed us to limit two types of biases. First, it allows the exclusion of false positives generated by bacteria at the limit of detection. Second, it limits the number of false positives generated by the random process of read selection during the rarefaction process. To test whether these bacteria indeed supported the floral cluster, we conducted an unsupervised hierarchical clustering analysis after excluding these 28 taxa. With this reduced dataset, the floral communities were no longer clustered (Fig. 4*C*). Second, to ensure that the removal of these 28 specific taxa, rather than the reduction in the dataset size, was responsible for the loss of the floral signal, we also generated 1,000 hierarchical clustering analyses with random exclusions of 28 taxa (not including one of our 28 candidate taxa) from each replication. Across these 1,000 analyses, the floral cluster was not eliminated, and this finding was supported in 96% of the 1,000 analyses (Fig. 4*D*). All 28 convergent and supported candidates were Proteobacteria, and 24 out of the 28 belonged to three groups and genera within the family Burkholderiaceae: *Burkholderia-Caballeronia-Paraburkholderia* group, *Pandorea*, and *Ralstonia* (*SI Appendix*, Table S1). Two candidates were Rhizobiaceae from the *Allorhizobium-Neorhizobium-Pararhizobium-Rhizobium* group. One was Enterobacteriaceae, which belongs to the genus *Arsenophonus*. One was a Beijerinckiaceae from the genus *Methylobacterium*. Apart from one Rhizobiaceae (ASV 1019), all the candidates were detected in floral samples not only from the plants grown under environmental conditions but also from the plants cultivated in the growth chamber. Because these 28 ASVs were repeatedly detected across samples, it is unlikely that sequencing errors generated these results (*SI Appendix*, Fig. S8). Furthermore, a thorough examination of the dataset suggests that these bacteria originated in the environment rather than from seeds or technical origins (see details in *SI Appendix*, *Text*). Finally, none of the candidate ASVs should stem from several 16S rRNA copies of the same bacterial taxa based on the observation that known 16S rRNA copies in genomes of plant-associated strains of Burkholderiaceae share 100% similarity within each strain and in the region of our amplicon (25) (see details in *SI Appendix*, *Text*).

### Bacteria Characteristic of Floral Communities.

To identify ASVs typical of the floral communities, we compared the IndVal metric for ASVs of communities of rosettes, leaves not in contact with soil, and flowers collected outside at the flowering stage (*Methods*). We conducted these analyses with a rarefied dataset and ASVs representing at least 0.1% of one of these communities. We implemented a permutation test procedure with a significance level of 4.0 e^−4^ for multiple-testing correction purposes (*Methods*). We identified 40 bacteria as being characteristic of floral communities. Among them, 25 were previously identified as being responsible for the convergence of floral communities (all the Burkholderiaceae and Rhizobiaceae ASV 1209; *SI Appendix*, Fig. S8 and Table S2). These bacteria responsible for the convergence of the floral community were significantly more abundant (24 out of 25) and significantly more frequently detected in floral communities (23 out of 25; *SI Appendix*, Fig. S8 and Table S2). Among the 15 remaining ASVs identified as being characteristic of flowers there were 10 Burkholderiaceae, 2 Rhizobiaceae, 1 Acetobacteriaceae, 1 Rhodanobacteraceae, and 1 Xanthomonadaceae (*SI Appendix*, Table S3).

## Discussion

Based on the composition of bacterial communities in environmental samples, it has been suggested that soil is an important source for bacterial migration toward the phyllosphere (5, 13–16). The present experiment suggests that at least a quarter of phyllosphere bacteria might have the capacity to colonize from soil, a proportion that increases to 75% when considering the top 10% of the most abundant taxa in these communities (Fig. 2*A* and *SI Appendix*, Figs. S1 and S2). With up to 73% of reads belonging to these taxa in the phyllosphere communities, bacteria able to reach the phyllosphere from soil can dominate this environment (*SI Appendix*, Fig. S3). Even if soil might not be the sole environmental origin for these taxa at the outside location, our results support that they can migrate without the help of the previously suggested dispersal factors wind, insect visits, and water splashing, which were not present for the plants cultivated in the growth chamber (17–20, 26). Consequently, bacterial migration from the soil toward the phyllosphere via surfaces, vessels, and gas convections is not unique to certain bacteria but is probably widely used by phyllosphere bacteria of herbaceous plant species with leaves close to the soil (27, 28).

In our study, the variation in the soil communities triggered changes in the rosette communities (Fig. 2*B*). This result is in agreement with Tkacz et al., who transplanted adult plants from one soil to another, resulting in changes in leaf communities (13). Our approach complements their findings by showing that even after the fixation of other edaphic conditions and without the induction of plant stress due to transplantations, variations in soil microbial communities alone are sufficient to induce changes in rosette communities. However, here, we found that the communities on leaves not in contact with the soil and flowers did not converge according to the soil communities in which the plants grew (Fig. 2*B*). This might be due to a relatively large contribution by factors associated with the air, random priority effects in leaf communities (29), and/or strong environmental filtering by these organs (5, 6, 16, 21). The present dataset did not allow us to dissect the relative contribution of these three contributing factors. However, the observation that soil ASVs that can reach the leaves away from soil have a higher relative abundance in this plant organ than in soil communities suggests the presence of environmental filtering by the leaves (*SI Appendix*, Fig. S3). Interestingly, the communities of leaves tended to form two clusters (clusters 1 and 2 in Fig. 4*A*). Cluster 1 was located apart from soil clusters and contained mainly bacterial communities of leaves away from soil and of rosettes that had grown in soils inoculated with community filtrates. Cluster 2 was nested within a soil community cluster and included bacterial communities of rosettes, the latter including leaves in direct contact with the soil. Despite the lack of statistical support, the clustering suggests that both source strength (i.e., the number of bacteria in soil) and soil-to-organ distance may impact the contribution of soil to the phyllosphere microbiota.

The floral communities were more similar to each other than to any other plant or soil community (Fig. 4 *A* and *B*). This convergence supports a strong selection of the surrounding microbial context by the floral environment. The convergence of floral communities from the growth chamber with those collected outside and the higher relative abundance of soil ASVs in floral communities suggest that soil bacteria can be filtered by the floral environment (Fig. 4*B* and *SI Appendix*, Fig. S3). Furthermore, this resemblance between outside and inside communities excludes convergence due to insect visits, which are rare in *A. thaliana* (6, 30, 31). Consequently, other floral characteristics, such as distinctive chemistry and microenvironmental conditions, must play an important role in this bacterial selection (5, 6, 32). The finding that almost all the bacteria responsible for this convergence of floral communities and that bacteria characteristic of these communities come from the same family (Burkholderiaceae) reinforces the presence of filtering that selects for certain bacteria with phylogenetically conserved functions (*SI Appendix*, Tables S1 and S3). This result is in accordance with the previous identification of phylogenetic conservatism in bacterial communities on leaves and flowers (7, 21).

Interestingly, Burkholderiaceae are enriched in disease-suppressive soils (33) and are known as soilborne pathogenic and beneficial bacteria in a wide range of plant hosts (33–37). The beneficial effects of these bacteria include drought stress tolerance in different plant species (38, 39) and plant growth promotion (40). They contribute to an increase in water content in plant tissues, which is critical in flowers to maintain floral architecture, and a proper fertilization process (38, 41). Culture-dependent and culture-independent approaches repeatedly detected members of *Burkholderia* and *Ralstonia* on flowers of different plant species, sometimes with high relative abundance (5, 9, 21, 42–45). These taxa have also been shown to colonize the inflorescences of grape vines by vessel migration from the rhizoplane (42). The other bacteria responsible for the convergence of the floral communities are also known to be associated with plants (46, 47). The relative abundances of two Rhizobiaceae (ASVs 1019 and 1209) and one Enterobacteriaceae (ASV 319) did not have strong differences between the flower and leaf communities, while *Methylobacterium* ASV 5, which is closely related to *Methylobacterium goesingense* and *Methylobacterium adhaesivum*, had a lower relative abundance in the flower community than in the leaf and soil communities (*SI Appendix*, Fig. S8).

The present study suggests that many soil bacteria could reach leaves and flowers of *A. thaliana* without the need for dispersal factors such as wind, insects, or water. Future experiments to investigate the capacity of bacteria to grow after reaching leaves and flowers from soil will help to better characterize the colonization of these organs from soil. The development of experimental systems closer to natural conditions regarding humidity, wind, or competition with bacteria coming from alternative sources will be particularly relevant to confirm the findings of the present study. In addition, it will be interesting to challenge our results with plant species whose leaves and flowers are farther from soil. Finally, synthetic communities will help to identify the routes used by these microorganisms. Our results also indicate that members of Burkholderiaceae, a plant-associated bacterial family, are part of a core floral community and are selected from the surrounding environment. It opens interesting perspectives to further investigate the causes leading to an enrichment of this bacterial family by the floral environment. Furthermore, the environmental filtering and enrichment of a specific family by the flowers of *Arabidopsis* call for similar experiments to determine if similar bacterial taxa are filtered at other locations and in other plant species. Additionally, the floral enrichment of this family makes them good candidates for intergenerational interactions with their hosts (i.e., vertical transmission). This mechanism is a strong driver for coevolution between hosts and symbionts and might explain many of the known positive and negative interactions between plants and *Burkholderia* relatives. Finally, reproducing the present experiment with different microbial variations (e.g., airborne microbiota) will help to further identify the most promising candidates with which to investigate plant–bacteria interactions.

## Methods

### Common-Garden Experiments and Collection of Samples.

We set up common-garden experiments with *A. thaliana* Col-0 at three different locations: one in a growth chamber at the Institute of Microbiology of Eidgenössische Technische Hochschule (ETH) Zurich and two in outside locations in the common-garden experimental facilities of ETH located at 47°22′47.6″N 8°32′54.3″E (Zurich location) and 47°27′01.2″N 8°40′56.3″E (Lindau location; Fig. 1). We chose *A. thaliana* because it is a widely used plant model that is commonly used to decipher the intricate relationships between the plant and its microbiome (3, 48–51). It also offers the application of many tools to design experiments to establish causal plant–microbe interactions (12, 52). In all these experiments, the treatment consisted of varying the soil communities without variations in other soil properties. To do so, we separately extracted two wild soil communities with a protocol that minimizes the extraction of soil chemicals. The first community came from an agricultural field (AG community; 47°24′21.1″N 8°30′27.4″E), and the second came from a nearby forest (FO community; 47°24′17.7″N 8°30′53.9″E). The extractions of the communities were performed on the same day, and the procedure was a modified version of the protocol by Hartman et al. (53). They consisted of sieving 16 L of each soil to remove stones. We used a large volume of soil to saturate the niches of our soil substitute (see below) with members of the extracted communities. This strategy aimed to limit potential invasions by exogenous environmental microorganisms. The 16 L of each of the natural soils was mixed in several batches for 1 min with a stick blender in phosphate-buffered saline (PBS) supplemented with 0.05% Tween to facilitate detachment of cells from soil particles. We collected the supernatants after 1.5 h to obtain larger particle sediments. Supernatants from the same soil were collected, pooled, and centrifuged at 3,000 × *g* for 10 min. Pellets containing microbial cells were resuspended in 0.5× Murashige and Skoog (MS) medium. The resulting solutions for the AG and FO communities were adjusted to pH 7 and used to inoculate our soil substitute. This soil substitute consisted of half washed and heat-sterilized calcined clay (Diamond Pro Calcined Clay Drying Agent; sterilization for 3 h at 200 °C) and half autoclaved vermiculite. Vermiculite was used to increase water retention in our soil substitute. The plant containers consisted of autoclaved plastic pots (130 × 130 mm) at the outside locations and gamma-irradiated microboxes (model OS140+ODS XXL; Microbox Combiness) in the growth chamber. Under sterile conditions, each container was filled with 200 mL of the soil substitute and inoculated with 85.7 mL of the MS medium containing the AG community or its filtrate or the FO community or its filtrate. We introduced the filtrates of the two inoculated MS media (using 0.22-μm filters) as soil treatments in each experiment to control for the potential impact of residual soil chemicals extracted during the procedure described above. Each container was planted with surface-sterilized full-sibling seeds of *A. thaliana* Col-0, which had been stratified for 7 d at 4 °C. Because the survival rates were expected to be lower at the outside locations, we placed several seeds at three separate spots in each pot and microbox (*SI Appendix*, Fig. S9). The sowing of seeds in the microboxes was conducted under sterile conditions in the laboratory, and the closed boxes were placed directly into the growth chamber. The sowing of the pots used at the outside locations was conducted at the experimental site, where they had been transported wrapped in sterile aluminum foil. In each common-garden experiment, each soil treatment included five replicates and were as follows: 1) soil inoculated with the AG community; 2) soil inoculated with the filtrate of the AG community; 3) soil inoculated with the FO community; and 4) soil inoculated with the filtrate of the FO community. Because we did not have the benefit of hindsight from previous comparable experiments, we could not exclude a homogenization of soil communities leading to the same soil communities in the AG and FO treatments at the end of the experiment. This might happen, for instance, if plants strongly select for specific soil communities or if the aerial communities at experimental sites erase initial AG and FO differences. To enable the interpretation of this potential failure, we added 10 pots without plants at each outside location. Five of the pots were inoculated with the AG community, and five were inoculated with the FO community. To verify the sterility of the seeds, we added three microboxes containing sterile 0.5× MS agar to the growth chamber, each of which was planted with several of our surface-sterilized seeds.

At the outside locations, we randomly shuffled the plant container positions twice a week to avoid spatial confounding effects in the analyses. Because growth chamber conditions were relatively stable through space and we wanted to limit the manipulation of the containers, which could generate cross-contamination between soil and aerial plant organs, we randomized the box positions once per week. The conditions in the growth chamber were set to favor plant flowering and consisted of a 16-h photoperiod at 22 °C and nighttime temperatures of 18 °C (both at 50% humidity). However, the humidity inside the microboxes was above 90%. Therefore, it was not necessary to water the plants in the growth chamber because the soil substitute remained wet throughout the experiment. At the outside locations, the pots were regularly watered when the soil substitute dried out. To avoid cross-contamination between the soil, leaves, and flowers, we filled the plant saucers with water and allowed the humidity to infiltrate the soil from the bottom. We first collected leaf and soil samples when the growth of rosettes was complete (the adult stage was identified as the start of inflorescence emergence). At this stage, we collected entire rosettes in both the growth chamber and Zurich location. Because of the large size of the plants in the growth chamber, we could also collect leaves that were not in contact with the soil (i.e., they were erect and at the center of the rosettes; *SI Appendix*, Fig. S9). At the outside locations, it was not possible to collect leaves that were not in contact with the soil because the plants were much smaller. We collected plant samples from only one of the three planted spots and left the remaining plants for the rest of the experiment. We also collected soil samples away from the plants. At the blooming stage, we collected rosettes, leaves not in contact with the soil, and inflorescences at both locations. At this stage, we collected soil samples from the surface as well as after mixing to better capture the bacterial richness in the pots, which is often spatially structured. For the growth chamber, we opened the containers and collected samples under sterile conditions to avoid contamination from the laboratory environment. All the samples were collected with gloves, flamed scissors, and forceps in 2-mL tubes. The samples were flash-frozen in liquid nitrogen immediately after collection and maintained at −80 °C until further processing.

### DNA Library Preparation, 16S Amplicon Sequencing, and Raw Data Processing.

The bacterial communities of all the samples collected from the common-garden experiments, five replicates of each original natural soil, and soil substitute collected just after their inoculation (starting points) were characterized by their 16S rRNA amplicon profiles (variable regions V5 to V7) sequenced with one 2 × 300 paired-end run on an Illumina MiSeq System. Because several processing steps involved treating the samples in batches before sequencing, we randomized the order in which they were treated. This procedure excluded the possibility of introducing technical biases as potential confounding factors in the statistical analyses (e.g., PCR plates). For each library preparation step, one master mix was made and used for all the samples. Sample preparation, genomic DNA extraction, and DNA library preparation were performed as described previously (21). The sole difference was that not two but all the negative control DNA extractions were processed and sequenced in addition to six empty samples introduced to the library after DNA extraction. The detailed procedure and associated statistics for demultiplexing, cleaning, and generating the relative abundance table from the raw data are available in Dataset S1. In short, we used USEARCH v11.0.667 (54) to filter reads with expected errors greater than one and to remove singletons. We defined ASVs based on 100% sequence similarity with the denoising strategy in USEARCH, which removes potential chimeras and potential sequencing errors. During this procedure, we excluded any sequence cluster with fewer than eight reads. Taxonomic identification was conducted using the SILVA database (reference no. 132_SSURef_Nr99) (55). The sequences without any hits in this database were searched against the Nucleotide database of the National Center for Biotechnology Information (NCBI) with BLAST (56). The ASVs identified as eukaryotic organisms were discarded from the dataset. Amplicon-metabarcoding approaches are subject to diverse artifacts, leading to the presence of reads in data that do not come from the bacteria present in the original biological samples (57). Microbial communities with low biomass are especially sensitive in this regard (58, 59). Lab and reagent contaminations are the most problematic biases, and no methods suggested in the literature have been shown to reliably eliminate them (58). The use of negative and mock community controls to try to exclude these artifacts from the interpretations of the results remains the gold standard. However, the systematic removal of bacteria detected in the negative control is not appropriate, as they can come from sample cross-contamination during library preparation (60), tag-switching events (61–64), or even real contamination but also be present in the sampled environment (58). In the present study, five ASVs detected in all negative controls were removed, as this is a good indication of contamination from DNA extractions or library preparation reagents. In addition, we conducted further analyses for all the ASVs of particular interest in the interpretation of our results by thoroughly examining their detection in all the negative controls and the mock Zymo community (*SI Appendix, Text*). All the samples with fewer than 1,000 reads were discarded before statistical analyses.

### Proportion of Phyllosphere Bacteria with the Potential to Colonize Plants from Soil.

To identify the proportion of bacterial taxa that were detected in the phyllosphere of plants collected outside and that have the potential to colonize from the soil, we compared the phyllosphere communities from the outside location with those from the growth chamber. Because the microboxes used in the growth chamber did not allow any external contamination other than that from the soil, air flows were strongly limited (but not gas-exchanged), water splashing did not occur in this environment, and the bacteria detected on the leaves and flowers in these containers passively or actively migrated from the soil via plant surfaces, vessels, or gas movements due to convection. To quantify ASVs shared between outside and inside plants, we combined the reads of plant samples collected outside into one pool and those of plant samples collected in the growth chamber into another. We conducted this pooling approach for AG and FO communities separately. To make the resulting pools comparable and test the robustness of the results, we repeatedly sampled the same number of reads in each pool using an incremental 100-read procedure and repeated absence–presence comparisons. We started from the number of reads in the smallest compared pool (outside or growth chamber) down to 100 reads (Datasets S2 and S3).

Because cross-contaminations and index-switching events can generate spurious presence, we conducted a sensitivity test consisting of the same comparisons as above after the transformation of the nonrarefied data with a presence threshold for all ASVs. All presences supported by fewer than three reads were transformed into zeros (absences). This threshold was defined from an exploration of the number of reads of ASVs not expected to be part of the community of the mock community control (Zymo) and still detected in this sample. The reasoning is that these reads were coming from cross-contaminations and index-switching events, and their average number was two reads. Because the sample size of this control was twice larger than the average number of reads per sample in the dataset (22,860 versus 10,866 reads), we estimated this value as being reasonably stringent.

We estimated the number of reads belonging to the ASVs that have the capacity to reach organ communities from soil in samples collected outside. We first rarefied all the sample sizes to the number of reads contained in the smallest plant sample collected outside (1,084 reads) and used the soil ASVs identified as being able to reach the phyllosphere after applying the presence threshold defined above.

### Differences in Soil Communities and Their Influence on Phyllosphere Community Divergence.

To test whether the soil communities were different in our experiment and whether these differences strongly influenced leaf and flower communities, we conducted unsupervised hierarchical clustering analyses that do not involve a priori assumptions in the definition and number of groups. We used the complete linkage algorithm, and the Bray–Curtis metric as a dissimilarity measure across samples. The goal was to see if the soil substitute communities collected outside were sorted according to the community used for their inoculation (i.e., AG or FO communities) and if the bacterial communities of the plants collected outside were sorted according to the community used to inoculate the soil in which the plants grew. For the plant organs, we conducted analyses with the following sample types separately: rosettes at the adult stage, rosettes at the flowering stage, leaves not in contact with the soil at the flowering stage, and inflorescences. Because the clustering according to soil communities could be influenced by residual chemical differences generated during the extraction of wild soil communities rather than microbial variations, we repeated the clustering analyses but with plant samples cultivated in soil substitutes inoculated with the filtrates of the inocula (see above *Common-Garden* *Experiments and* *Collection of* *Samples* section). To make all the analyses comparable, we rarefied all the sample sizes to the number of reads contained in the smallest plant sample collected outside (1,084 reads). To test whether the observed clusters were created not by chance but biology, we generated 1,000 bootstrapped datasets and repeated the clustering analysis with each of them. We visually summarized these 1,000 analyses with a majority-rule consensus dendrogram in which all the clusters being supported in at least half of the analyses were represented, whereas clusters supported in less than 50% of analyses were collapsed. For each represented cluster, we calculated the percentage of analyses in which it was supported. Because the rarefaction procedure consists of randomly sampled reads, it can also generate clusters supported by chance. To control for this additional bias, we generated 1,000 rarefactions for each dataset. As previously described, we conducted hierarchical clustering analyses with all these independent data, summarized the results with a majority-rule consensus dendrogram, and calculated the percentages of recovery for each cluster. We considered a cluster to be statistically supported when it was supported in both the bootstrapped and sequential rarefaction analyses.

### Convergence of Bacterial Communities and Identification of the Responsible Bacterial Taxa.

To measure the convergence of the bacterial communities in specific organs, we conducted unsupervised hierarchical clustering analyses following the same procedure as above, but with soil and plant samples collected outside all included in the same analysis. The communities of a specific organ were considered convergent when they formed a well-supported cluster, that is, when they were more similar to each other than to any other type of community.

To identify the bacteria underlying this pattern (i.e., higher similarity among communities on a specific plant organ in comparison with those of other plant parts or soil), we applied the following procedures. 1) We performed 1,000 rarefactions of the dataset at 1,084 reads; 2) the relative abundances of each bacterial taxon were used separately to conduct unsupervised hierarchical clustering analyses across the 1,000 rarefied datasets; and 3) each taxon was considered a candidate if its data generated a cluster of communities for the organ of interest in all 1,000 rarefied datasets. This conservative approach, which consisted of excluding any potential ASV candidate if its data did not support the cluster of interest in all the rarefied datasets, allowed us to limit two types of biases. First, it allows the exclusion of false positives generated by bacteria at the limit of detection. Second, it limits the number of false positives generated by the random process of read selection during rarefaction. Once the list of candidates was identified with this procedure, we tested whether withdrawing these specific taxa from communities erased the clustering pattern previously observed. Finally, for the latter test, we tested whether it was not the reduction in the size of the dataset which was responsible for the loss of clustering. To do so, we repeated the hierarchical clustering analyses after 1,000 random exclusions of the same number of taxa, not including one of the identified candidates. If the candidate taxa and not the reduction in the size of the dataset were responsible for the clustering, the organ cluster was expected to be supported in these latter analyses. We identified several Burkholderiaceae as being responsible for floral community convergences. Because these bacteria are known to be common extraction kit and laboratory contaminants (43, 58, 59, 65, 66), we thoroughly explored the dataset to ensure that these bacteria were not technical or seed contamination (see details in *SI Appendix*, *Text*, Figs. S10 and S11, and Tables S4–S7 and Dataset S4). The use of ASVs in the present study requires caution due to the presence of multiple 16S rRNA copies in bacterial cells. If these different copies have small differences, they will belong to different ASVs. We ensured that the 16S rRNA copies in Burkholderiaceae shared 100% similarity in the region of our amplicon (see details in *SI Appendix*, *Text*).

The environmental sources of the bacterial taxa that are responsible for the convergence of the organ communities could be the aerial environment, rain, biotic interactions, or soil. To test whether the convergence involves the selection of soil bacteria, we reconducted the hierarchical clustering analysis with plant samples collected outside during the flowering stage in addition to all the plant samples grown in the inoculated soil substitutes in the growth chamber and collected during the flowering stage. If the clustering of communities involves environmental filtering by organs of soil communities, then the growth chamber samples should be nested within the cluster of the same type of samples collected outside.

### Identification of Bacteria Characteristic of Floral Communities.

In order to identify the ASVs that characterize floral communities, we used the IndVal statistic, which is the product of the mean relative abundance of a taxon in a group of samples compared with all groups in the study (component A) and the relative frequency of detection of the taxon in the samples of the group of interest (component B) (67). A taxon being characteristic of the floral communities is defined as being more relatively abundant and more frequently detected in this environment than by chance. We rarefied the dataset of plant communities collected outside at the flowering stage at 1,084 reads. We calculated the IndVal statistic and its two components A and B for all bacteria representing at least 0.1% of the total number of reads of one of the communities. We compared the three following groups: rosettes (20 samples), leaves not in contact with the soil (20 samples), and flowers (19 samples). We identified the ASVs for which the IndVal metric was the highest for the group of floral communities. For these latter taxa, we conducted a randomization testing procedure (10,000 shuffling iterations) to calculate *P* values and estimated if the IndVal and A and B components were higher than expected under the null hypotheses of the ASV not being indicative of the floral group, not more relatively abundant in floral communities, and not more often detected in floral samples than by chance under the null hypothesis. We conducted these tests for 142 ASVs and considered a *P* value of 4.0 e^−4^ to be significant (0.05 significance level divided by 142) to correct for multiple testing.

For all ASVs identified as being characteristics of floral communities and not already investigated in the clustering analyses, we thoroughly explored the dataset to ensure that these bacteria were not technical or seed contaminations (see details in *SI Appendix*, *Text* and Tables S4–S7 and Dataset S4).

## Supplementary Material

Supplementary File

Supplementary File

Supplementary File

Supplementary File

Supplementary File

Supplementary File

## Data Availability

The raw sequencing data reported in this article are available in the European Nucleotide Archive repository (https://www.ebi.ac.uk/ena), accession no. PRJEB47294 (68). All the other data generated and used in the present study are included in this published article and its supporting information. All the codes used to demultiplex the raw sequencing data and conduct quality filtering, chimera filtering, ASV definition, and annotation can be found in Dataset S1. All the code used for statistical analyses can be found in Datasets S2 and S3. The ASV and metadata tables can be found in Dataset S5.
