# Supplementary material for: Capacity of soil bacteria to reach the phyllosphere and convergence of floral communities despite soil microbiota variation
Source: Proc Natl Acad Sci U S A. 2021 Oct 7;118(41):e2100150118. doi: 10.1073/pnas.2100150118 (PMC8521660; doi:10.1073/pnas.2100150118)
Supplement: Supplementary File [file pnas.2100150118.sapp.pdf]

PNAS
www.pnas.org

SI Appendix for

Soil bacteria migration to leaves and flowers and floral community convergence

Julien Massoni[1-2 *], Miriam Bortfeld-Miller[1], Alex Widmer[3], Julia A. Vorholt[1 *]

[1] Institute of Microbiology, Department of Biology, ETH Zurich, Vladimir-Prelog-Weg 1-5/10, 8093 Zurich, Switzerland

[2] Center for Adaptation to a Changing Environment, ETH Zurich, 8092 Zurich, Switzerland

[3] Institute of Integrative Biology, ETH Zurich, Zurich, Switzerland

* Julien Massoni, Julia Vorholt

Email: jmassoni@gmail.com, jvorholt@ethz.ch

This PDF file includes:

Supplementary text
Figures S1 to S11
Tables S1 to S7
Legends for Datasets S1 to S5

Other supplementary materials for this manuscript include the following:

Datasets S1 to S5

SI1: Data exploration to identify potential seed contaminations and biased identifications of bacteria responsible for the convergence of floral communities

Potential contaminations from seeds

To detect the presence bacteria coming from a potential incomplete sterilization of seeds, we first explored the diversity detected in leaves and flowers of plants grown in sterile 0.5X MS agar and three microboxes (see Methods). These samples had reads associated with different bacteria, which could come from an incomplete sterilization process, contaminations during experimental manipulations, library preparation (1), or tag switching events (2–5) (SI appendix, Table S4). All seeds used in this experiment came from the same plant individual and were sterilized together. Consequently, seed-bacterial contaminations are expected to generate congruent patterns across the three microboxes, unlike other sources of contaminations. The community data of the second and third boxes were in accordance with this assumption (SI appendix, Table S4), whereas communities of the first box had a much higher richness despite its lower number of reads (8,960 in first microbox, 32,890 in the second, and 35,870 in the third). In this first microbox, 68 taxa had more than 5 reads in at least one of the plant organs, whereas the second and third boxes had 11 and 12, respectively (SI appendix, Table S4). The bacteria in the first microbox not being detected in the two other boxes are likely originating from a contamination during experimental manipulation. The second and third microboxes shared eight bacteria with more than five reads. Except for one, these eight taxa were also detected in the first microbox (SI appendix, Table S4). Among them ASVs 3 and 8 were also detected in the original soil, starting points (see Methods), and in the soil of pots placed at the outside location without plants (see Methods; SI appendix, Table S5). This presence in soil samples which were not in contact with our sterile seeds suggested that these bacteria were either coming from another source of contamination or were seed contaminants being also present in the soil communities used for the experiment. In addition, ASVs 13 and 4092 were not detected in the remaining samples of the library leading to their exclusion from all analyses conducted in the present study. At the end, four *Bacillus* ASVs might be seed contaminations included in the analyses of the present study (SI appendix, Table S6). Because of the large richness detected in the present study (6,010 taxa), the effect of these four taxa on the results is negligible. For this reason and because these ASVs could explain biological patterns, we did not exclude them.

Potential contaminations of microboxes from laboratory contaminants

During the entire experiment, we opened the microboxes from the growth chamber at two different occasions. A first time to collect plant and soil samples at the plant-adult stage, and a second time to collect samples at the flowering stage. All microboxes were opened under sterile conditions, but lab contaminations could still happen. To check for this eventuality, we identified the 264 ASVs which were detected in the growth chamber but not detected in the original soils, inoculums, or starting points. Because natural soil communities harbor extremely rich microbial communities (6) even large depth of sequencing systematically fail to detect all bacteria present in the original samples. For this reason, these 264 ASVs were probably present in the inoculum communities and not detected, rather than being lab contaminants. Their absence with more than five reads in sterile plant samples grown in microboxes filled with sterile 0.5X MS agar and opened at the same time as all other microboxes, further support their origin from the inoculum rather than from lab contaminations (SI Appendix, Table S4).

Checking that taxa responsible for floral community convergence and being characteristic of floral communities are not seed or technical contaminations

Because Burkholderiaceae are known to be common extraction-kit and lab contaminants (7–11), we thoroughly explored the dataset to ensure that these bacteria were not technical or seed contaminations. Apart from one *Ralstonia* (ASV 3), one *Burkholderia* (ASV 8), and one *Phyllobacterium* (ASV 156), all bacteria identified as being filtered by the floral environment are virtually absent from samples of control plants, and the Zymo community (see section "Seed sterilization" above for details; SI Appendix, Table S7). The few reads being sometimes found in these samples probably originated from cross contaminations during library preparation (1), and tag switching events (2–5). In addition, most of them were detected in communities of original soils, of starting points, and of soil samples collected from pots outside without plant cultivated in (See methods; SI Appendix, Table S5). These result support their environmental rather than laboratory or seed origin. The ASVs 3 and 8 were detected in virtually all controls, but also in communities of the original soils, and other soil samples (SI Appendix, Tables S5 and S7). In the original soil communities, they were among the first half most abundant ASVs (SI Appendix, Fig. S10). It suggests that even if they partly came from a lab contamination, they were probably also present in original soils. These original soil communities contained large biomass and diverse bacteria. Because lab contaminations are expected to be at low biomass, their abundance should be overwhelmed by data from bacteria of the original soil and not being detected (or being in the lower part of abundance distribution). Furthermore, if these two bacteria partly contributed to the convergence of the floral communities, their removal from the data did not erase the hierarchical clustering signal of floral communities (SI Appendix, Fig. S11). The ASV 156 was detected in negative controls of DNA extractions, but not in other controls and in the Zymo community (SI Appendix, Table S7). Its detection in all communities of the original soils, and other soil samples also suggests that even if it partly came from a lab contamination, it was probably also present in original soils (SI Appendix, Tables S5). This later bacterium was not detected as being responsible for the convergence of floral communities.

ASV definition and potential biases

The presence of several 16S rRNA copies in species of Burkholderiaceae might be an important bias when ASVs are used as taxonomical units (12). If the sequences of these copies differ in bacterial cells, several of our ASVs might come from the same bacteria. Among ASVs being responsible for the floral convergence several belonged to the genera *Burkholderia* and *Ralstonia* (SI Appendix, Table S1). We downloaded from the rrnDB v5.6 (12) the sequences of 16S rRNA copies of all species of these two genera present in this database and known to be plant associated. Within each species, all 16S rRNA copies shared 100% similarity in the region of our amplicon (Dataset S4). Consequently, we can safely consider each of the ASVs belonging to these genera to be different bacterial taxa.

Sequencing errors is a second bias to be considered when ASVs are defined. The denoise algorithm aims to correct for this type of bias. Furthermore, the repeatedly detection of our 28 candidates across samples supports their biological origin rather than error-containing sequences which are not expected to have high prevalence across samples (13).

REFERENCES

1. D. C. Murray, M. L. Coghlan, M. Bunce, From benchtop to desktop: important considerations when designing amplicon sequencing workflows. *PLoS One* **10**, e0124671 (2015).

2. M. C. Nelson, H. G. Morrison, J. Benjamino, S. L. Grim, J. Graf, Analysis, optimization and verification of Illumina-generated 16S rRNA gene amplicon surveys. *PLoS One* **9**, e94249 (2014).

3. M. Kircher, S. Sawyer, M. Meyer, Double indexing overcomes inaccuracies in multiplex sequencing on the Illumina platform. *Nucleic Acids Res.* **40**, e3 (2012).

4. P. Esling, F. Lejzerowicz, J. Pawlowski, Accurate multiplexing and filtering for high-throughput amplicon-sequencing. *Nucleic Acids Res.* **43**, 2513–2524 (2015).

5. I. B. Schnell, K. Bohmann, M. T. P. Gilbert, Tag jumps illuminated - reducing sequence-to-sample misidentifications in metabarcoding studies. *Mol. Ecol. Resour.* **15**, 1289–1303 (2015).

6. T. W. Crowther, *et al.*, The global soil community and its influence on biogeochemistry. *Science* **365**, eaav0550 (2019).

7. H. Toju, R. L. Vannette, M.-P. L. Gauthier, M. K. Dhami, T. Fukami, Priority effects can persist across floral generations in nectar microbial metacommunities. *Oikos* **127**, 345–352 (2018).

8. A. Glassing, S. E. Dowd, S. Galandiuk, B. Davis, R. J. Chiodini, Inherent bacterial DNA contamination of extraction and sequencing reagents may affect interpretation of microbiota in low bacterial biomass samples. *Gut Pathog.* **8**, 24 (2016).

9. M. C. de Goffau, *et al.*, Recognizing the reagent microbiome. *Nat. Microbiol.* **3**, 851–853 (2018).

10. S. J. Salter, *et al.*, Reagent and laboratory contamination can critically impact sequence-based microbiome analyses. *BMC Biol.* **12**, 1–12 (2014).

11. J. Rosindell, L. J. Harmon, OneZoom: A Fractal Explorer for the Tree of Life. *PLoS Biol.* **10**, e1001406 (2012).

12. S. F. Stoddard, B. J. Smith, R. Hein, B. R. K. Roller, T. M. Schmidt, rrnDB: Improved tools for interpreting rRNA gene abundance in bacteria and archaea and a new foundation for future development. *Nucleic Acids Res.* **43**, D593–D598 (2015).

13. B. J. Callahan, P. J. McMurdie, S. P. Holmes, Exact sequence variants should replace operational taxonomic units in marker-gene data analysis. *ISME J.* **11**, 2639–2643 (2017).

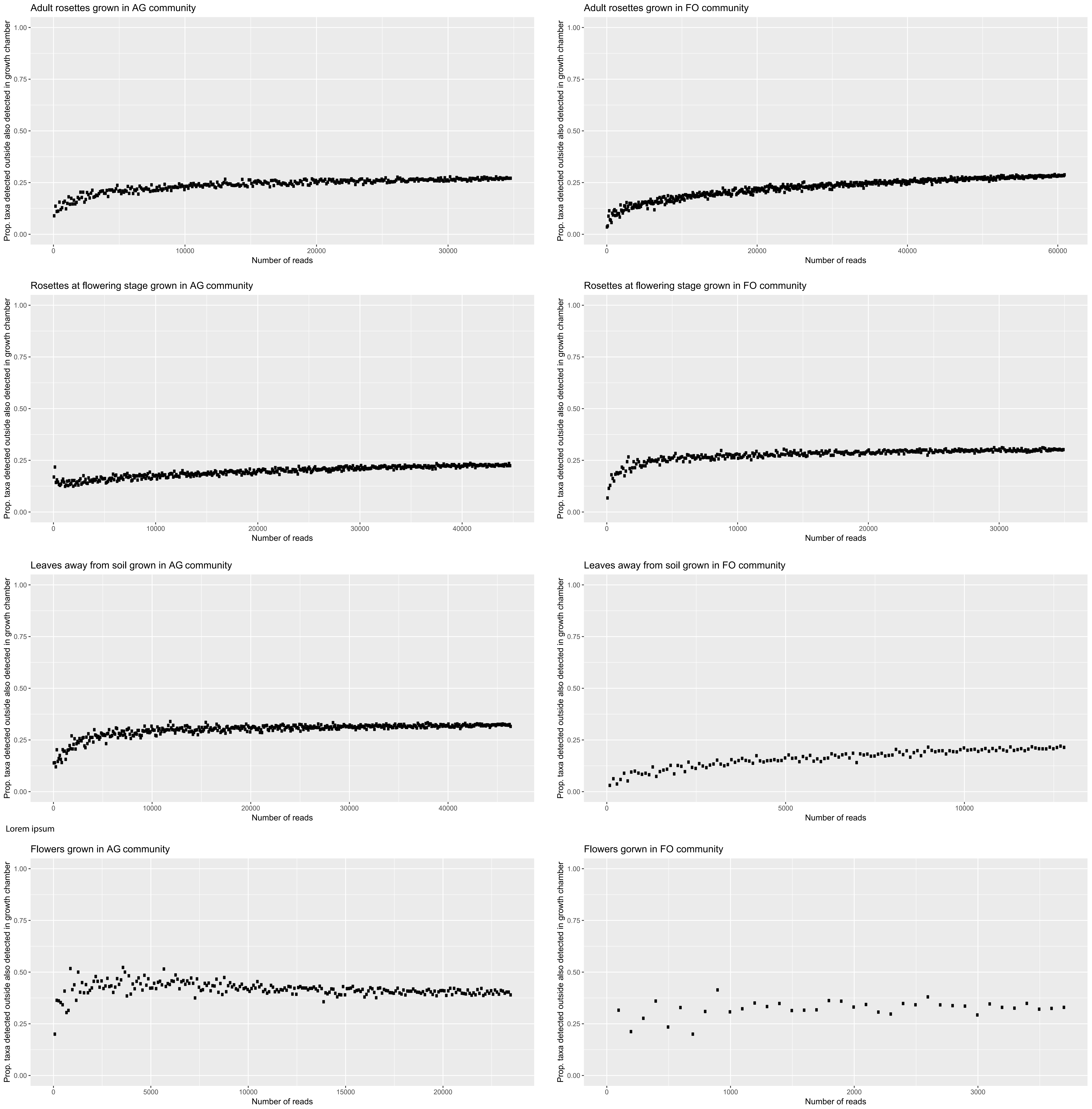

Figure S1: Proportion of ASVs in each plant organ detected outside and in the growth chamber. Abbreviation: prop., proportions.

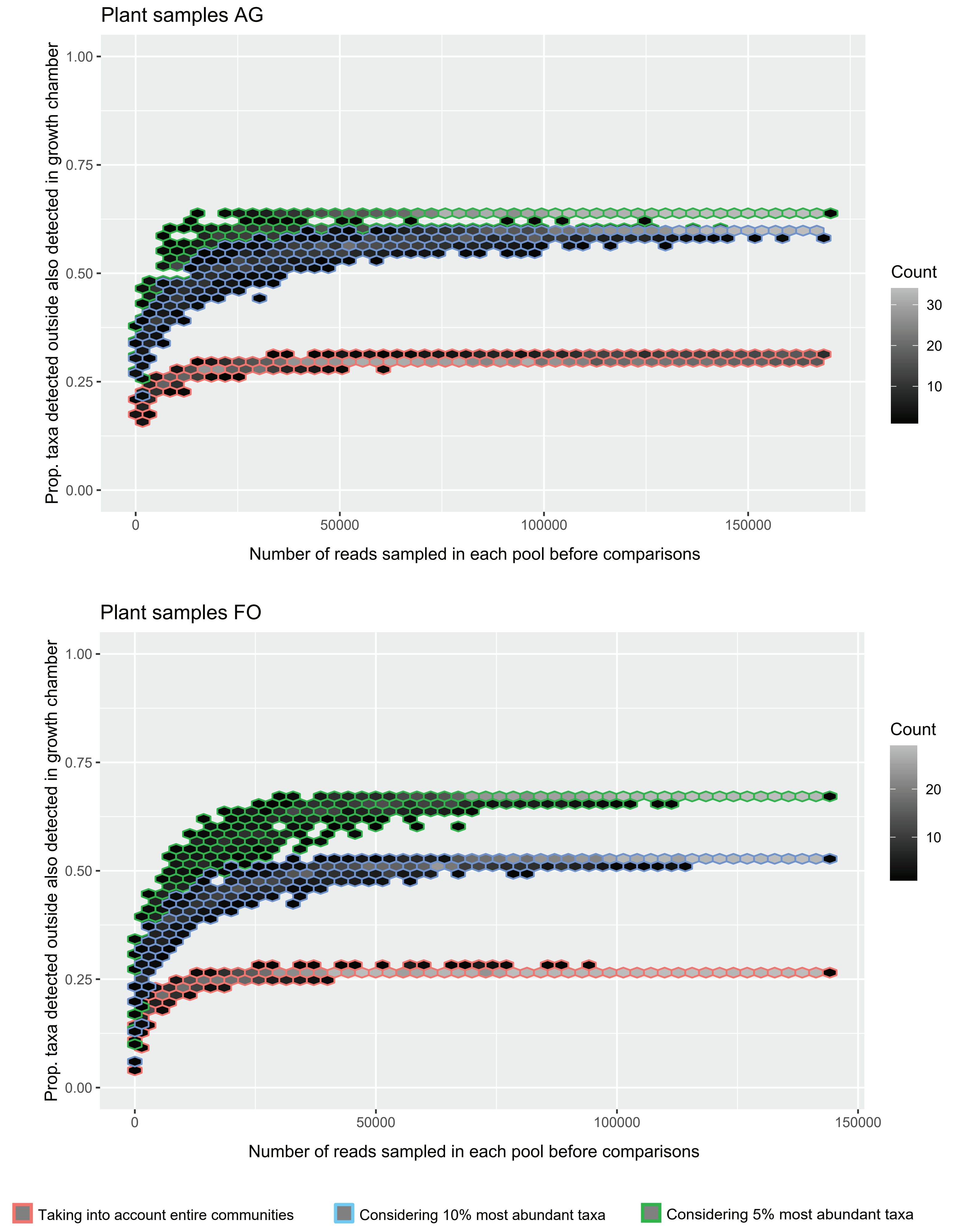

Figure S2: Hexagonal heatmap of the proportion of ASVs detected in phyllosphere at outside location also detected in phyllosphere communities in the growth chamber after definition of a presence threshold at three reads. All samples collected outside were combined in one pool, and those collected inside in another before comparisons.

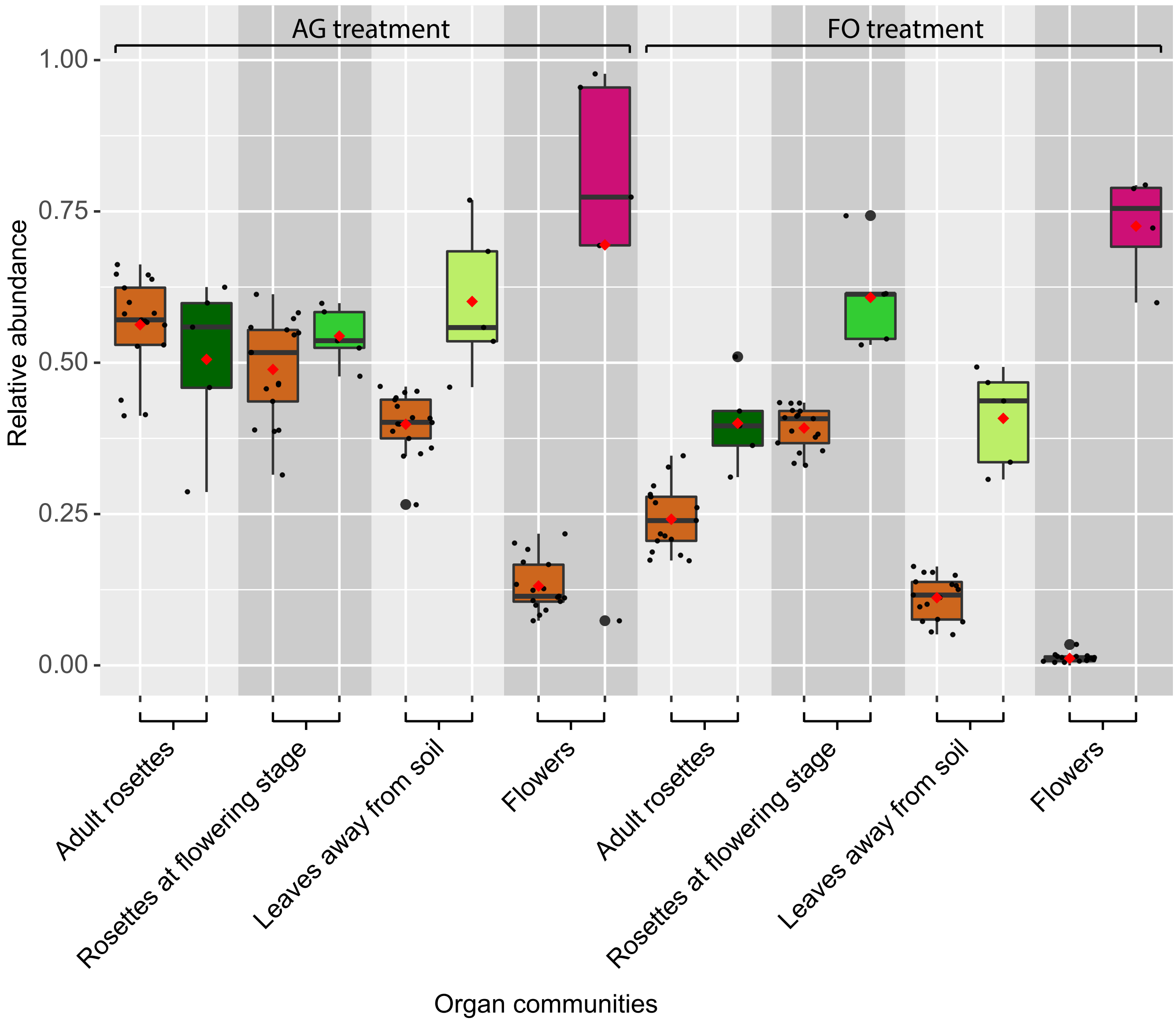

Figure S3: Box plots of the relative abundances of the ASVs that can reach plant organs of the phyllosphere from soil. For each organ, their relative abundances are presented in the soil communities (maroon box plots), and in the organ communities (green and pink box plots).

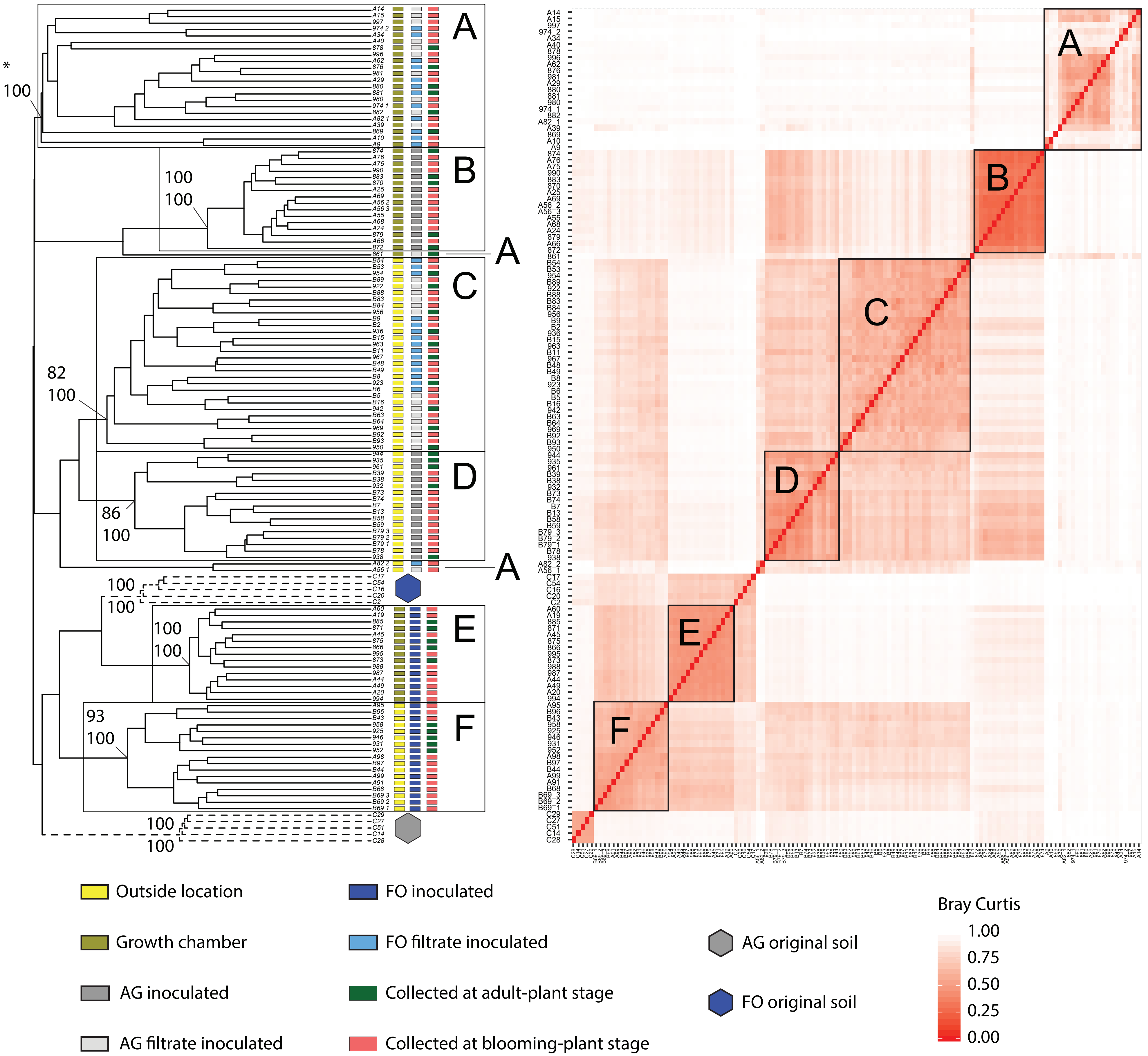

Figure S4: Unsupervised hierarchical clustering of soil communities. The Bray-Curtis metric was used as a measure of dissimilarity and was calculated from rarefied data. The first panel is the resulting dendrogram, and the second panel is the corresponding heatmap. The nodes of the clusters discussed in the text (A to F) are labeled with the percentages of the independent hierarchical clustering analyses that support them; the analyses were conducted with 1,000 bootstrapped datasets (upper number) and 1,000 rarefied datasets (lower number). Each tip of the dendrogram is labeled with the location of collection, the soil treatment, and the plant developmental stage at which the samples were collected. The two hexagons represent the samples of original soils.

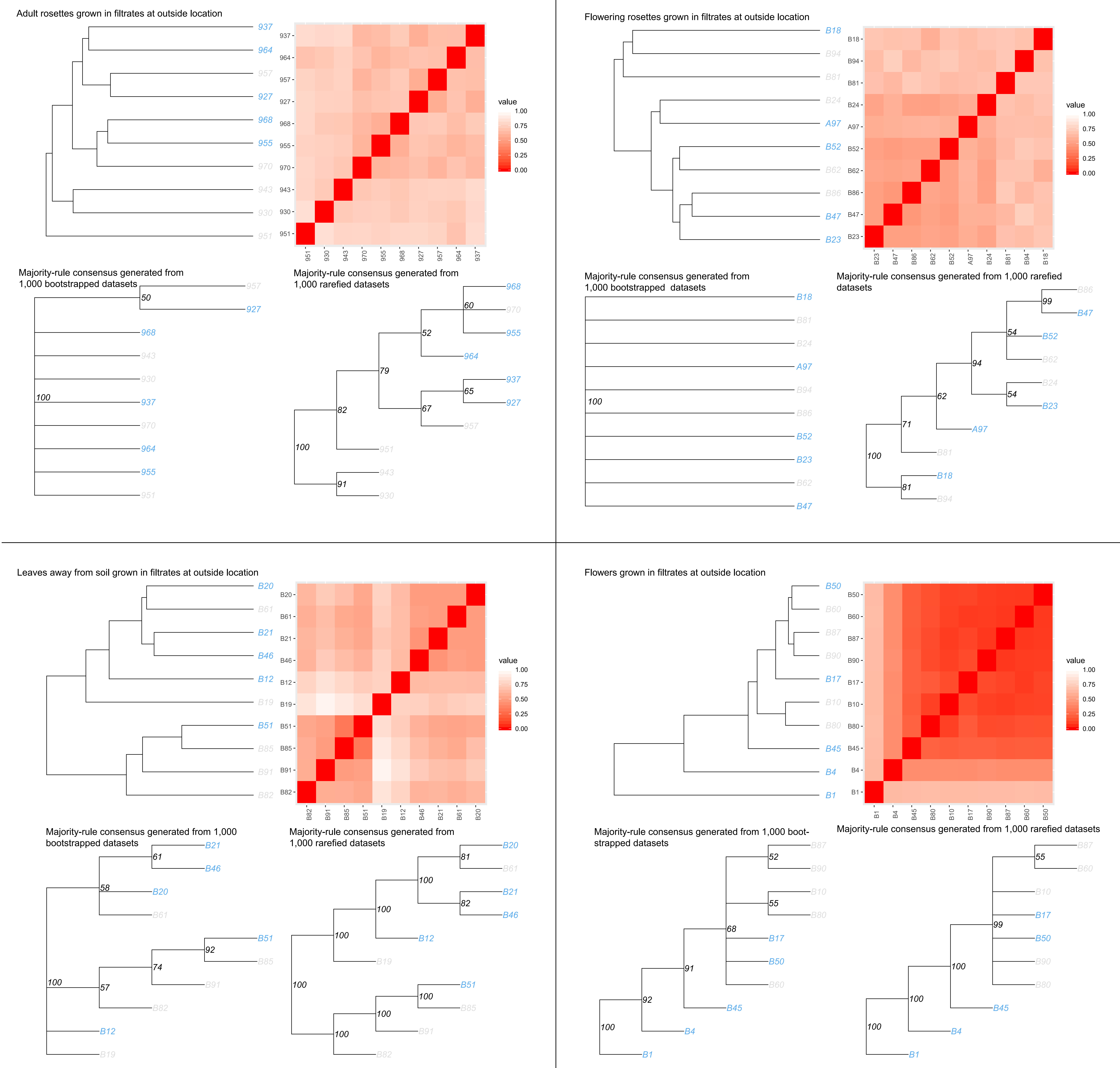

Figure S5: Unsupervised hierarchical clustering of phyllosphere communities collected outside and grown in soil substitutes inoculated with filtrates of AG and FO inocula. The communities are identified by their sample number. The data are all rarefied according to the number of reads of the smallest plant sample collected outside, and the sample differences were calculated with the Bray-Curtis metric. Each panel consists of a dendrogram and a heatmap generated from one rarefied dataset and two majority rule consensuses. The first consensus summarizes hierarchical clustering analyses based on 1,000 bootstrapped datasets, and the second summarizes hierarchical clustering analyses based on 1,000 rarefied datasets. The nodes are labeled with the percentages of recovery of each cluster across these iterative analyses. The sample numbers are colored according to the filtrate used to inoculate the soil in which the plants grew (gray: AG community, blue: FO community).

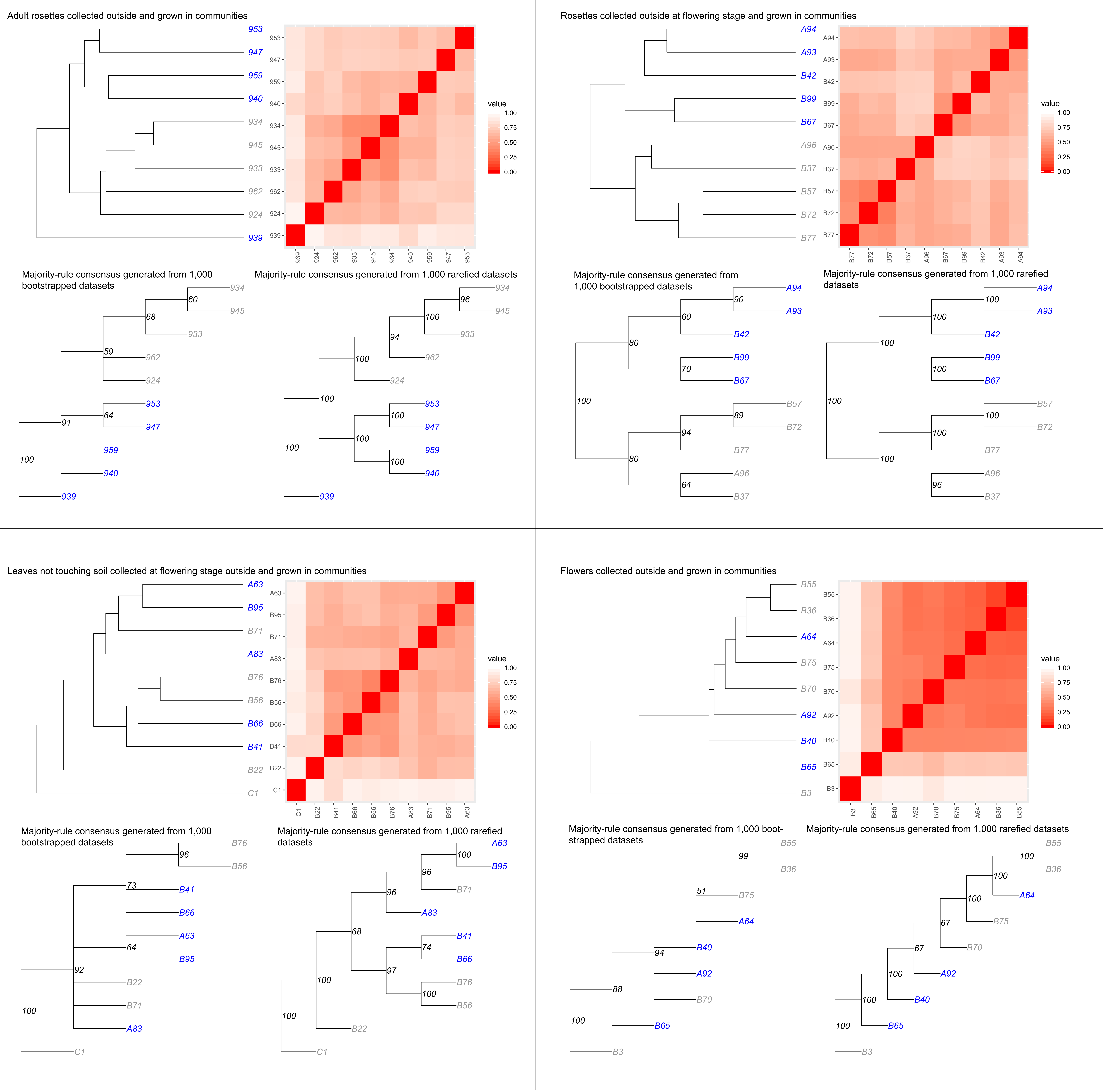

Figure S6: Unsupervised hierarchical clustering of phyllosphere communities collected outside, using the Bray-Curtis metric as a measure of dissimilarity. The communities are identified by their sample number. The data are rarefied according to the number of reads of the smallest sample included in each analysis. Each panel consists of a dendrogram and a heatmap generated from one rarefied dataset and two majority rule consensuses. The first panel summarizes hierarchical clustering analyses based on 1,000 bootstrapped datasets, and the second summarizes hierarchical clustering analyses based on 1,000 rarefied datasets. The nodes are labeled with the percentage of recovery of each cluster across these iterative analyses. The sample numbers are colored according to the community used to inoculate the soil in which the plants were cultivated (gray: AG community, blue: FO community).

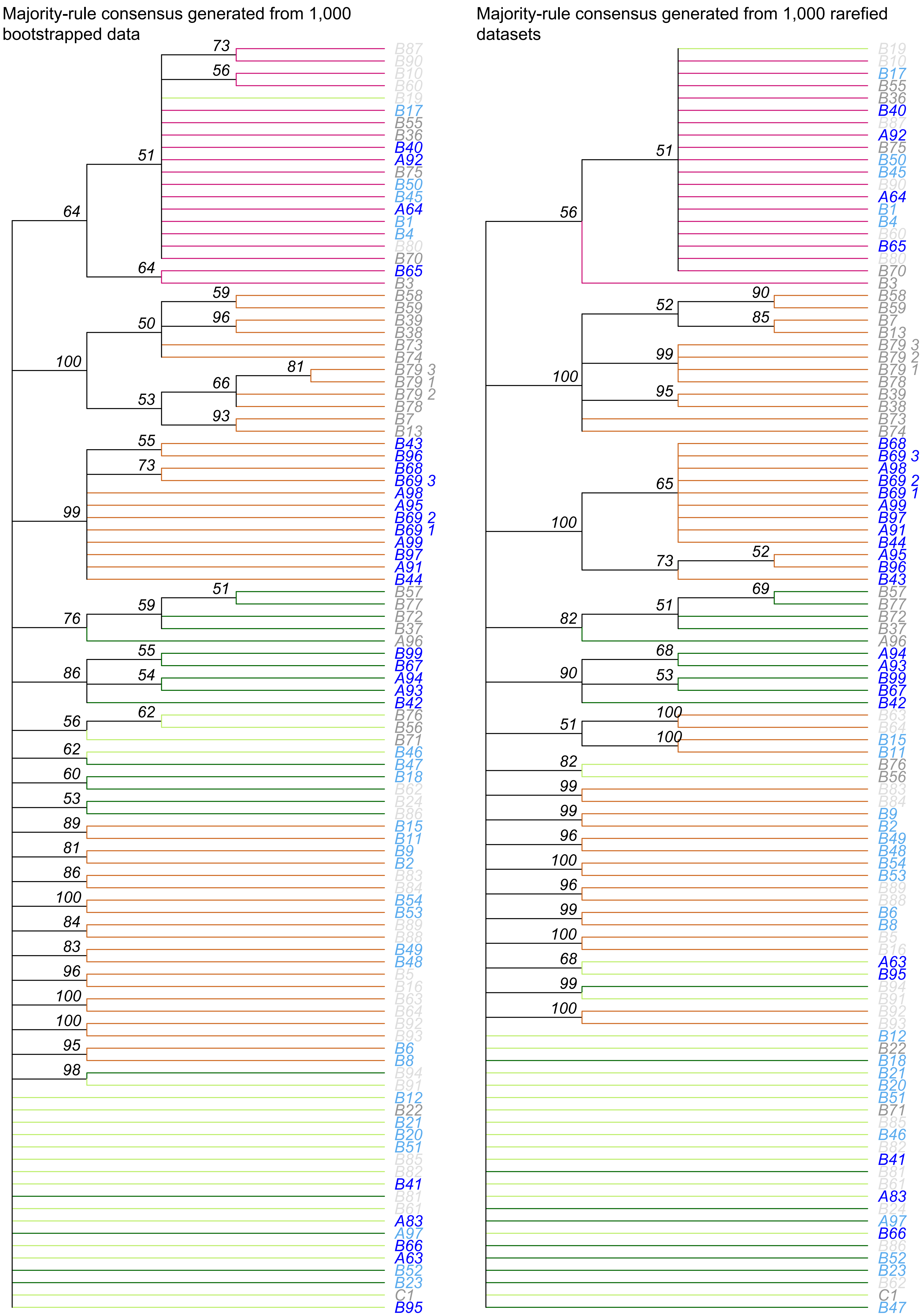

Figure S7: Unsupervised hierarchical clustering analyses based on a binary Bray-Curtis metric of all the communities from the plant and soil samples collected during the blooming stage from the plants grown outside. The communities are identified by their sample number. The data were rarefied according to the number of reads of the smallest plant sample. The first dendrogram is a majority rule consensus generated from 1,000 bootstrapped datasets. The second dendrogram is a majority rule consensus generated from 1,000 rarefied datasets. The nodes are labeled with the percentages of recovery of each cluster across these iterative analyses. The colors of the branches and sample numbers follow the codes used in Figure 4.

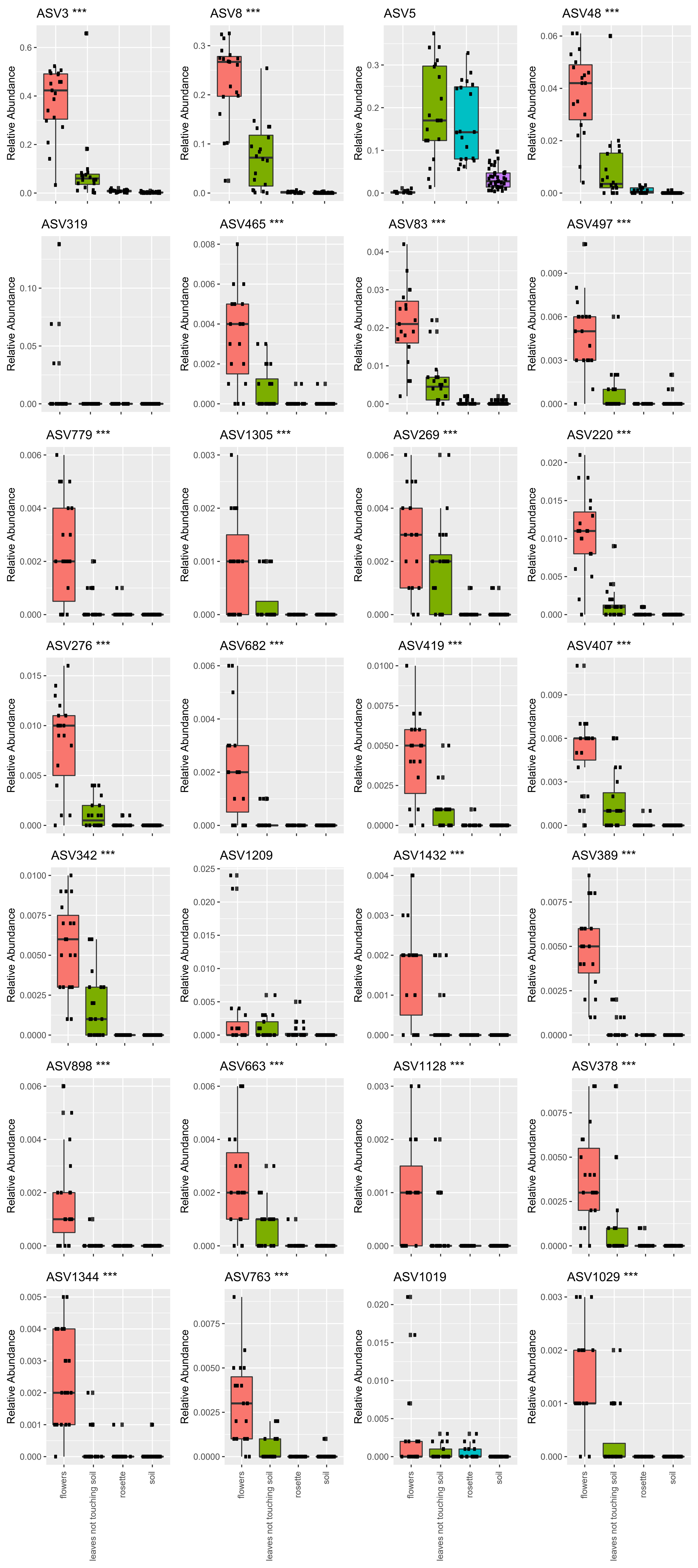

Figure S8: Box plots of the relative abundances (in rarefied data) of the ASVs responsible for the convergence of floral communities in flowers, leaves not in contact with the soil, rosettes, and soil communities collected outside during the flowering stage. Significance at the 4.0 e-4 level for a relative abundance being higher in flowers than in leaves not touching the soil and in rosettes is indicated by asterisks after the ASV names. P-values were calculated from a permutation test with the A component of the IndVal statistics.

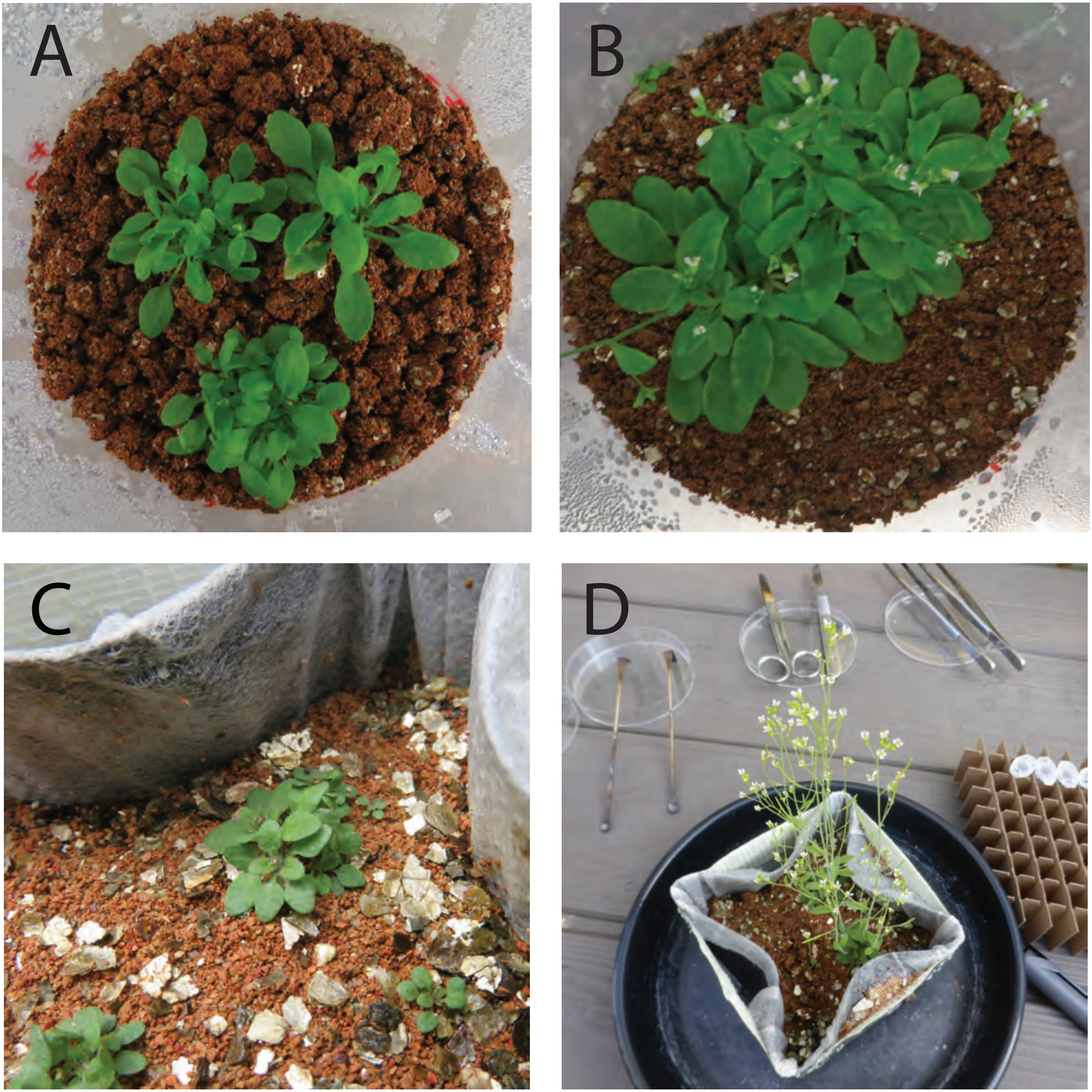

Figure S9: Examples of individual plants from the outside location and growth chamber at the adult and blooming stages. A, Adult plants in the growth chamber. B, Blooming plants in the growth chamber. C, Adult plants at the outside location. D, Blooming plants at the outside location.

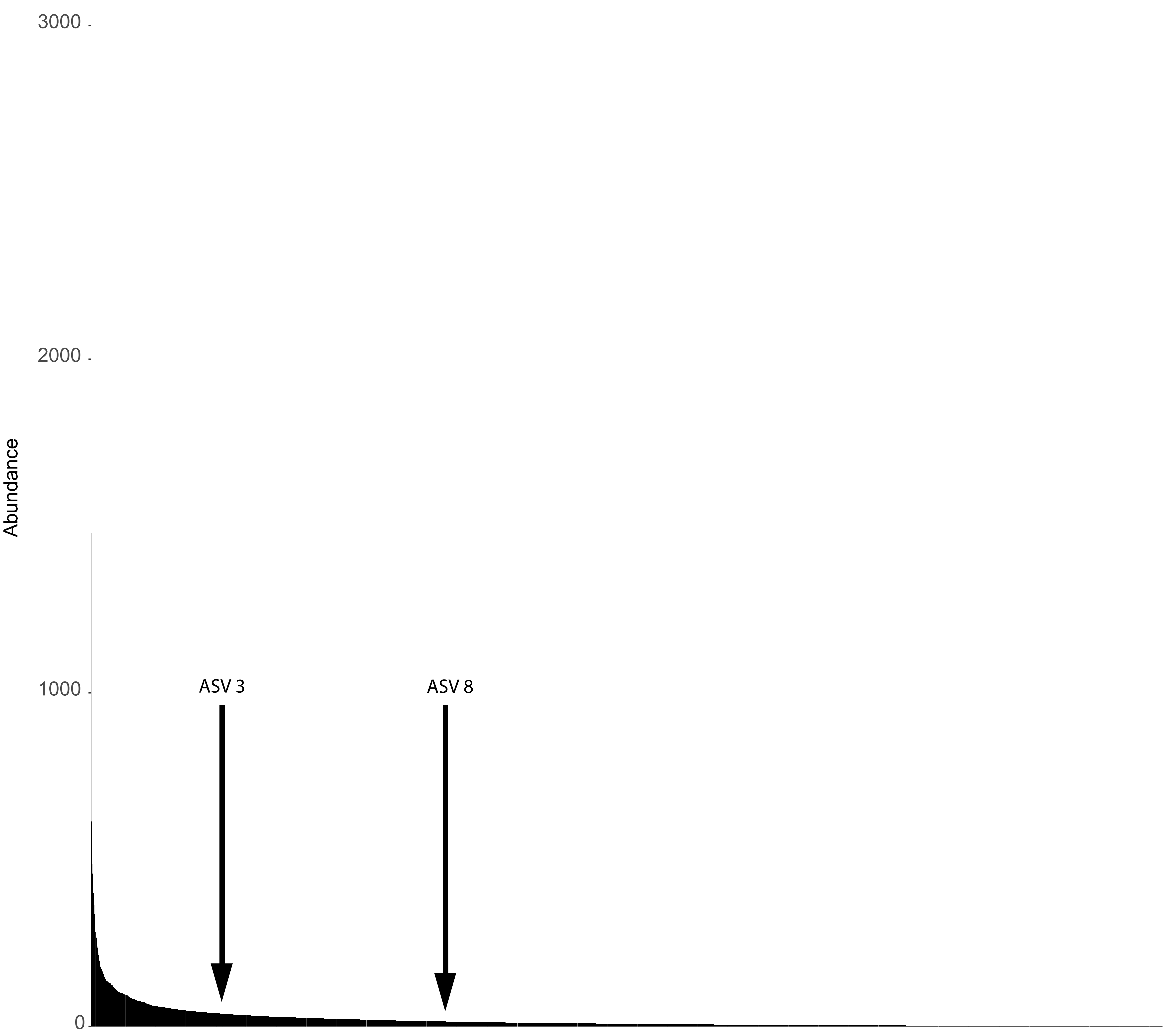

Figure S10: Bacterial composition of the original soil communities, with the positions of ASVs 3 and 8 indicated.

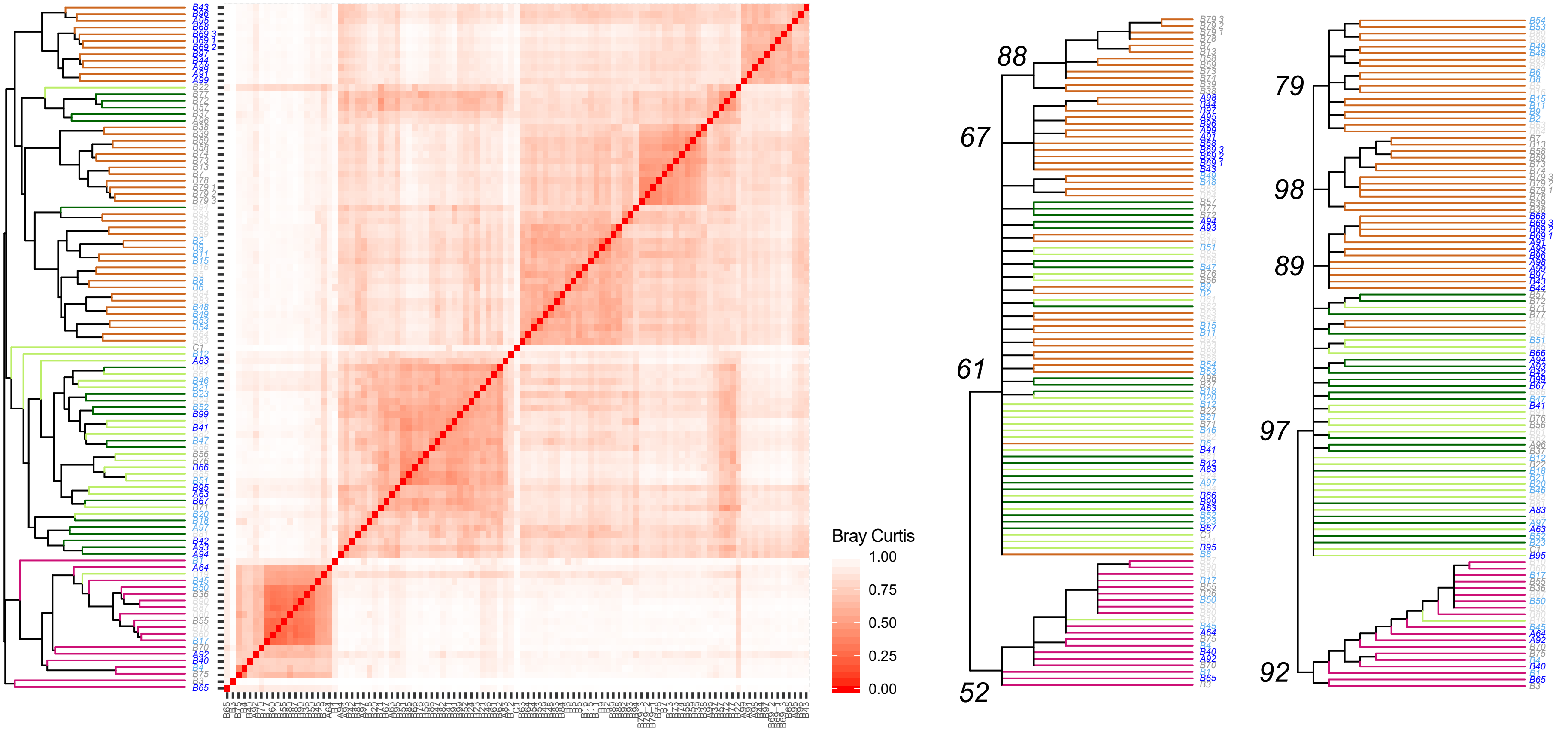

Figure S11: Unsupervised hierarchical clustering of phyllosphere communities collected outside after the removal of ASVs 3 and 8 from the data. The Bray-Curtis metric is used as a measure of dissimilarity. The communities are identified by their sample number. The data are rarefied according to the number of reads of the smallest sample included in this analysis. The first dendrogram and a heatmap were generated from one rarefied dataset. The second dendrogram summarizes hierarchical clustering analyses based on 1,000 bootstrapped datasets, and the third dendrogram summarizes analyses based on 1,000 rarefied datasets. The nodes are labeled with the percentage of recovery of each cluster across these iterative analyses. The sample numbers are colored according to the community used to inoculate the soil in which the plants were cultivated (gray: AG community, blue: FO community).

Taxon	Phylum	Class	Order	Family	Genus	Species	BLAST identifications at 100% similarity in NCBI data bases
ASV5	Proteobacteria	Alphaproteobacteria	Rhizobiales	Beijerinckiaceae	Methylobacterium	unidentified	M. goesingense / M. adhaesivum
ASV1209	Proteobacteria	Alphaproteobacteria	Rhizobiales	Rhizobiaceae	Allorhizobium-Neorhizobium-Pararhizobium-Rhizobium	unidentified	NA
ASV1019	Proteobacteria	Alphaproteobacteria	Rhizobiales	Rhizobiaceae	Allorhizobium-Neorhizobium-Pararhizobium-Rhizobium	unidentified	NA
ASV378	Proteobacteria	Gammaproteobacteria	Betaproteobacteriales	Burkholderiaceae	Burkholderia-Caballeronia-Paraburkholderia	Burkholderia sp. M27-VN8-1W	NA
ASV407	Proteobacteria	Gammaproteobacteria	Betaproteobacteriales	Burkholderiaceae	Burkholderia-Caballeronia-Paraburkholderia	Burkholderia sp. SY237	NA
ASV342	Proteobacteria	Gammaproteobacteria	Betaproteobacteriales	Burkholderiaceae	Burkholderia-Caballeronia-Paraburkholderia	Burkholderia sp. SY237	NA
ASV663	Proteobacteria	Gammaproteobacteria	Betaproteobacteriales	Burkholderiaceae	Burkholderia-Caballeronia-Paraburkholderia	Burkholderia sp. SY237	NA
ASV1029	Proteobacteria	Gammaproteobacteria	Betaproteobacteriales	Burkholderiaceae	Burkholderia-Caballeronia-Paraburkholderia	Burkholderia sp. SY237	NA
ASV48	Proteobacteria	Gammaproteobacteria	Betaproteobacteriales	Burkholderiaceae	Burkholderia-Caballeronia-Paraburkholderia	uncultured	NA
ASV1344	Proteobacteria	Gammaproteobacteria	Betaproteobacteriales	Burkholderiaceae	Burkholderia-Caballeronia-Paraburkholderia	uncultured Burkholderia sp.	NA
ASV8	Proteobacteria	Gammaproteobacteria	Betaproteobacteriales	Burkholderiaceae	Burkholderia-Caballeronia-Paraburkholderia	unidentified	NA
ASV465	Proteobacteria	Gammaproteobacteria	Betaproteobacteriales	Burkholderiaceae	Burkholderia-Caballeronia-Paraburkholderia	unidentified	Paraburkholderia nodosa / P. ribeironis / P. ferrariae
ASV269	Proteobacteria	Gammaproteobacteria	Betaproteobacteriales	Burkholderiaceae	Burkholderia-Caballeronia-Paraburkholderia	unidentified	NA
ASV763	Proteobacteria	Gammaproteobacteria	Betaproteobacteriales	Burkholderiaceae	Burkholderia-Caballeronia-Paraburkholderia	unidentified	NA
ASV497	Proteobacteria	Gammaproteobacteria	Betaproteobacteriales	Burkholderiaceae	Pandoraea	uncultured bacterium	NA
ASV779	Proteobacteria	Gammaproteobacteria	Betaproteobacteriales	Burkholderiaceae	Ralstonia	metagenome	NA
ASV3	Proteobacteria	Gammaproteobacteria	Betaproteobacteriales	Burkholderiaceae	Ralstonia	Ralstonia	Ralstonia solanacearum
ASV389	Proteobacteria	Gammaproteobacteria	Betaproteobacteriales	Burkholderiaceae	Ralstonia	Ralstonia	NA
ASV1128	Proteobacteria	Gammaproteobacteria	Betaproteobacteriales	Burkholderiaceae	Ralstonia	Ralstonia	NA
ASV220	Proteobacteria	Gammaproteobacteria	Betaproteobacteriales	Burkholderiaceae	Ralstonia	Ralstonia solanacearum	NA
ASV276	Proteobacteria	Gammaproteobacteria	Betaproteobacteriales	Burkholderiaceae	Ralstonia	Ralstonia solanacearum	NA
ASV682	Proteobacteria	Gammaproteobacteria	Betaproteobacteriales	Burkholderiaceae	Ralstonia	Ralstonia solanacearum	Ralstonia solanacearum
ASV419	Proteobacteria	Gammaproteobacteria	Betaproteobacteriales	Burkholderiaceae	Ralstonia	uncultured bacterium	NA
ASV1305	Proteobacteria	Gammaproteobacteria	Betaproteobacteriales	Burkholderiaceae	Ralstonia	unidentified	NA
ASV83	Proteobacteria	Gammaproteobacteria	Betaproteobacteriales	Burkholderiaceae	unidentified	unidentified	Pelomonas saccharophila
ASV319	Proteobacteria	Gammaproteobacteria	Enterobacteriales	Enterobacteriaceae	Arsenophonus	uncultured Arsenophonus sp.	uncultured Arsenophonus sp.
ASV1432	unidentified	unidentified	unidentified	unidentified	unidentified	unidentified	Burkolderiaceae*
ASV898	unidentified	unidentified	unidentified	unidentified	unidentified	unidentified	Ralstonia*

* for these two ASVs, we did not find any BLAST hit with 100% identity, but we reported the genera of best hits because we did not have any taxonomical information from the SILVA database.

Table S1: Taxonomic annotations from the SILVA database of the 28 ASVs responsible for the convergence of floral communities. In the last column, we added the species from the BLAST hits with 100% similarity with our sequences. NA: not applicable, as no BLAST hits with 100% similarity with our sequences were found.

	Observed IndVal	P-value IndVal	Observed A component of IndVal	P-value of A component	Observed B component of IndVal	P-value of B component
ASV3*	77.96	0	0.78	0	1.00	0.433
ASV8*	73.98	0	0.74	0	1.00	0
ASV178	38.50	0	0.46	0	0.84	0
ASV48*	79.74	0	0.80	0	1.00	0
ASV465*	76.60	0	0.81	0	0.95	0
ASV298	47.89	0	0.70	0	0.68	0
ASV83*	77.38	0	0.77	0	1.00	0
ASV1190	62.80	0	0.80	0	0.79	0
ASV497*	82.17	0	0.87	0	0.95	0
ASV779*	68.42	0	0.93	0	0.74	0
ASV1305*	41.80	0	0.88	0	0.47	0
ASV269*	49.24	0	0.62	0	0.79	0
ASV220*	85.43	0	0.85	0	1.00	0
ASV1138	51.32	0	0.81	0	0.63	0
ASV276*	87.10	0	0.87	0	1.00	0
ASV682*	80.34	0	0.90	0	0.89	0
ASV1104	63.16	0	0.92	0	0.68	0
ASV419*	80.44	0	0.90	0	0.89	0
ASV407*	81.69	0	0.86	0	0.95	0
ASV1364	42.86	0	0.68	0	0.63	0
ASV342*	73.68	0	0.78	0	0.95	0
ASV443	39.68	0	0.54	0	0.74	0
ASV1432*	59.33	0	0.94	0	0.63	0
ASV389*	87.38	0	0.87	0	1.00	0
ASV1198	33.08	0	0.79	0	0.42	0
ASV898*	59.87	0	0.88	0	0.68	0
ASV901	51.91	0	0.90	0	0.58	0
ASV1214	29.15	0	0.69	0.0012	0.42	2.00E-04
ASV663*	66.50	0	0.74	0	0.89	0
ASV1128*	53.19	0	0.84	0	0.63	0
ASV378*	81.70	0	0.86	0	0.95	0
ASV1344*	65.50	0	0.78	0	0.84	0
ASV763*	73.07	0	0.87	0	0.84	0
ASV1029*	54.45	0	0.86	0	0.63	0
ASV1777	33.49	0	0.91	0	0.37	0
ASV1995	37.89	0	0.90	0	0.42	0
ASV2871	32.75	0	0.89	0	0.37	0
ASV1209*	34.65	1.00E-04	0.73	0.001	0.47	0.0027
ASV156	25.51	2.00E-04	0.69	2.00E-04	0.37	0.001
ASV250	28.45	2.00E-04	0.54	2.00E-04	0.53	5.00E-04

Table S2: Significant observed IndVal metrics at the 4.0 e-4 significance level, calculated for the floral-sample group. A and B components are also presented with their respective pvalues. ASVs labelled with an asterisk were those identified as being responsible for the convergence of floral communities in hierarchical clustering analyses.

Taxon	Phylum	Class	Order	Family	Genus	Species	BLAST identifications in NCBI databases
ASV1214	Proteobacteria	Alphaproteobacteria	Acetobacterales	Acetobacteraceae	Acidocella	unidentified	
ASV298	Proteobacteria	Gammaproteobacteria	Betaproteobacteriales	Burkholderiaceae	unidentified	unidentified	
ASV1138	Proteobacteria	Gammaproteobacteria	Betaproteobacteriales	Burkholderiaceae	Ralstonia	Ralstonia solanacearum	
ASV1104	Proteobacteria	Gammaproteobacteria	Betaproteobacteriales	Burkholderiaceae	Burkholderia-Caballeronia-Paraburkholderia	uncultured Burkholderia sp.	
ASV1198	Proteobacteria	Gammaproteobacteria	Betaproteobacteriales	Burkholderiaceae	Ralstonia	metagenome	
ASV1777	Proteobacteria	Gammaproteobacteria	Betaproteobacteriales	Burkholderiaceae	Cupriavidus	unidentified	
ASV1995	Proteobacteria	Gammaproteobacteria	Betaproteobacteriales	Burkholderiaceae	Burkholderia-Caballeronia-Paraburkholderia	uncultured	
ASV2871	Proteobacteria	Gammaproteobacteria	Betaproteobacteriales	Burkholderiaceae	Burkholderia-Caballeronia-Paraburkholderia	unidentified	
ASV156	Proteobacteria	Alphaproteobacteria	Rhizobiales	Rhizobiaceae	Phyllobacterium	unidentified	
ASV443	Proteobacteria	Alphaproteobacteria	Rhizobiales	Rhizobiaceae	Mesorhizobium	unidentified	
ASV178	Proteobacteria	Gammaproteobacteria	Xanthomonadales	Rhodanobacteraceae	Dyella	Dyella	
ASV1190	unidentified	unidentified	unidentified	unidentified	unidentified	unidentified	Burkholderia sp.*
ASV1364	unidentified	unidentified	unidentified	unidentified	unidentified	unidentified	Burkholderia*
ASV901	unidentified	unidentified	unidentified	unidentified	unidentified	unidentified	Ralstonia pickettii*
ASV250	Proteobacteria	Gammaproteobacteria	Xanthomonadales	Xanthomonadaceae	Stenotrophomonas	unidentified	

* for these ASVs, we reported the first identified taxa with the BLAST search, because we did not retreived information from the Silva database. Percentages of identity were superior to 95%.

Table S3: Taxonomic annotations from the SILVA database of the ASVs identified as being characteristic of the floral communities and not being presented in the Table S1. In the last column, we added the first taxonomical identification from a BLAST search with NCBI data bases for ASVs for which no information was available in the Silva database.

Microbox 1	Rosettes at adult stage (sample 887)	Rosettes at flowering stage (sample A1)	Flowers (sample A2)	Total abundance across all samples of the box
ASV3	1190	1491	2171	4852
ASV8	33	135	1580	1748
ASV48	13	157	193	363
ASV13	1	5	288	294
ASV83	39	44	135	218
ASV220	42	23	42	107
ASV276	27	22	30	79
ASV269	26	34	11	71
ASV389	24	16	28	68
ASV298	20	8	12	40
ASV419	9	12	19	40
ASV342	2	2	34	38
ASV378	0	17	21	38
ASV497	0	3	31	34
ASV407	0	4	28	32
ASV25	25	0	4	29
ASV78	11	17	0	28
ASV682	7	11	9	27
ASV101	14	5	6	25
ASV465	0	4	20	24
ASV898	9	7	7	23
ASV64	22	0	0	22
ASV5	7	0	14	21
ASV763	0	4	17	21
ASV1712	11	8	0	19
ASV671	0	2	17	19
ASV779	3	10	5	18
ASV663	0	2	16	18
ASV57	15	0	2	17
ASV501	11	5	1	17
ASV5424	16	0	0	16
ASV1128	3	8	4	15
ASV479	15	0	0	15
ASV203	11	3	0	14
ASV250	8	6	0	14
ASV543	0	13	1	14
ASV1305	8	2	4	14
ASV1104	0	8	6	14
ASV760	6	7	1	14
ASV1576	4	9	0	13
ASV807	1	0	11	12
ASV130	12	0	0	12
ASV304	1	7	4	12
ASV357	7	5	0	12
ASV19	0	10	1	11
ASV1432	0	0	11	11
ASV5206	0	0	11	11
ASV6	9	0	1	10
ASV62	0	0	10	10
ASV443	0	0	10	10
ASV901	3	2	5	10
ASV1995	1	3	6	10
ASV1138	5	4	0	9
ASV1344	0	2	7	9
ASV1858	1	8	0	9
ASV1198	3	4	1	8
ASV665	0	0	7	7
ASV1364	0	0	7	7
ASV773	0	4	3	7
ASV1029	0	2	5	7
ASV4524	7	0	0	7
ASV58	6	0	0	6
ASV1214	0	6	0	6
ASV811	0	6	0	6
ASV5096	0	6	0	6
ASV61	0	3	2	5
ASV56	5	0	0	5
ASV1775	5	0	0	5
ASV178	1	2	1	4
ASV22	4	0	0	4
ASV109	4	0	0	4
ASV1190	0	1	3	4
ASV314	0	4	0	4
ASV249	0	0	4	4
ASV396	0	4	0	4
ASV1268	0	4	0	4
ASV645	0	4	0	4
ASV2757	0	0	4	4
ASV3802	1	3	0	4
ASV1664	1	0	3	4
ASV5323	4	0	0	4
ASV2871	0	0	4	4
ASV4	3	0	0	3
ASV21	0	2	1	3
ASV11	0	0	3	3
ASV59	0	1	2	3
ASV191	0	3	0	3
ASV63	2	0	1	3
ASV726	0	1	2	3
ASV54	0	2	1	3
ASV212	0	0	3	3
ASV180	3	0	0	3
ASV427	2	0	1	3
ASV4783	3	0	0	3
ASV3520	0	0	3	3
ASV1076	0	3	0	3
ASV1827	0	0	3	3
ASV1964	0	3	0	3
ASV2051	1	0	2	3
ASV1437	0	0	3	3
ASV10	0	2	0	2
ASV14	1	1	0	2
ASV34	1	0	1	2
ASV49	0	1	1	2
ASV586	0	2	0	2
ASV24	1	0	1	2
ASV96	0	0	2	2
ASV154	2	0	0	2
ASV68	0	0	2	2
ASV234	0	0	2	2
ASV67	0	2	0	2
ASV292	0	2	0	2
ASV156	0	0	2	2
ASV35	1	1	0	2
ASV877	0	0	2	2
ASV102	0	2	0	2
ASV706	0	0	2	2
ASV1307	0	0	2	2
ASV1292	0	0	2	2
ASV1145	0	0	2	2
ASV892	0	0	2	2
ASV3206	0	0	2	2
ASV4148	0	2	0	2
ASV4774	0	0	2	2
ASV370	0	0	1	1
ASV69	1	0	0	1
ASV106	0	0	1	1
ASV258	1	0	0	1
ASV159	1	0	0	1
ASV320	0	0	1	1
ASV50	0	0	1	1
ASV7	0	1	0	1
ASV582	0	0	1	1
ASV417	1	0	0	1
ASV172	1	0	0	1
ASV89	0	1	0	1
ASV42	0	0	1	1
ASV1736	1	0	0	1
ASV182	1	0	0	1
ASV155	0	1	0	1
ASV373	0	0	1	1
ASV237	0	0	1	1
ASV527	1	0	0	1
ASV121	0	1	0	1
ASV705	0	0	1	1
ASV162	1	0	0	1
ASV483	1	0	0	1
ASV283	1	0	0	1
ASV406	0	1	0	1
ASV361	0	1	0	1
ASV872	0	0	1	1
ASV455	0	1	0	1
ASV1031	0	0	1	1
ASV227	0	1	0	1
ASV374	0	0	1	1
ASV1003	0	1	0	1
ASV431	0	1	0	1
ASV387	0	1	0	1
ASV5027	1	0	0	1
ASV470	0	0	1	1
ASV464	1	0	0	1
ASV558	1	0	0	1
ASV1425	1	0	0	1
ASV518	0	1	0	1

Microbox 2	Rosettes at adult stage (sample 862)	Rosettes at flowering stage (sample A6)	Flowers (sample A7)	Flowers (sample A8)	Total abundance across all samples of the box
ASV13	10339	4998	8606	7648	31591
ASV671	32	30	132	132	326
ASV665	33	24	126	131	314
ASV807	18	18	109	133	278
ASV1827	5	3	32	40	80
ASV3	1	2	26	37	66
ASV4092	4	8	21	27	60
ASV8	1	3	20	23	47
ASV27	0	8	0	0	8
ASV5	0	2	5	1	8
ASV127	0	0	0	5	5
ASV4	0	2	1	0	3
ASV6	1	1	1	0	3
ASV479	0	2	0	1	3
ASV22	1	1	0	0	2
ASV48	0	1	0	1	2
ASV107	0	2	0	0	2
ASV292	2	0	0	0	2
ASV94	0	0	1	1	2
ASV741	0	0	2	0	2
ASV63	0	0	2	0	2
ASV406	0	0	2	0	2
ASV250	0	0	2	0	2
ASV218	0	2	0	0	2
ASV269	1	0	0	1	2
ASV220	0	1	0	1	2
ASV419	0	0	0	2	2
ASV5408	0	1	1	0	2
ASV368	0	0	2	0	2
ASV10	0	0	0	1	1
ASV12	0	0	1	0	1
ASV140	0	0	1	0	1
ASV41	0	0	1	0	1
ASV144	0	1	0	0	1
ASV58	0	0	1	0	1
ASV24	0	0	1	0	1
ASV209	0	0	1	0	1
ASV101	1	0	0	0	1
ASV33	1	0	0	0	1
ASV45	1	0	0	0	1
ASV87	0	0	0	1	1
ASV117	0	0	1	0	1
ASV23	0	0	1	0	1
ASV266	0	0	1	0	1
ASV83	0	0	0	1	1
ASV121	1	0	0	0	1
ASV156	1	0	0	0	1
ASV149	0	0	1	0	1
ASV223	0	0	0	1	1
ASV290	0	0	0	1	1
ASV35	0	1	0	0	1
ASV543	0	0	1	0	1
ASV350	0	1	0	0	1
ASV304	0	0	0	1	1
ASV877	0	0	0	1	1
ASV682	0	0	0	1	1
ASV194	0	0	0	1	1
ASV331	0	0	0	1	1
ASV403	0	0	1	0	1
ASV381	0	0	1	0	1
ASV1815	1	0	0	0	1
ASV536	0	0	1	0	1
ASV991	0	0	1	0	1
ASV239	0	1	0	0	1
ASV700	0	0	1	0	1
ASV625	0	1	0	0	1
ASV584	0	0	1	0	1
ASV1206	0	1	0	0	1
ASV1371	1	0	0	0	1
ASV2646	1	0	0	0	1
ASV4723	0	0	1	0	1
ASV610	0	0	0	1	1
ASV4118	0	0	1	0	1
ASV3831	0	0	0	1	1
ASV2217	0	0	1	0	1
ASV1319	0	0	0	1	1
ASV6115	1	0	0	0	1
ASV6059	0	0	0	1	1
ASV1398	0	0	1	0	1
ASV1227	0	1	0	0	1
ASV2058	0	1	0	0	1
ASV2080	0	1	0	0	1
ASV1491	0	0	1	0	1
ASV1766	0	0	0	1	1
ASV2229	0	1	0	0	1
ASV2154	0	0	1	0	1
ASV4200	0	0	1	0	1
ASV2666	0	0	1	0	1
ASV4183	0	0	1	0	1
ASV1324	0	0	1	0	1
ASV2745	0	0	0	1	1
ASV4523	0	0	1	0	1
ASV3773	0	0	1	0	1
ASV4017	0	0	1	0	1
ASV3591	0	1	0	0	1
ASV4776	0	0	1	0	1
ASV4024	0	0	0	1	1
Total number of reads	10447	5120	9122	8201	32890

Microbox 3	Rosettes at adult stage (sample 897)	Rosettes at flowering stage (sample A4)	Flowers (sample A5)	Total abundance across all samples of the box
ASV13	11068	13726	9637	23363
ASV807	84	138	144	282
ASV665	93	135	121	256
ASV671	78	136	123	259
ASV1827	17	27	30	57
ASV4092	23	20	26	46
ASV3	9	12	18	30
ASV8	2	9	4	13
ASV14	8	0	4	4
ASV30	0	8	0	8
ASV10	3	2	1	3
ASV76	5	0	0	0
ASV6	0	2	3	5
ASV17	4	0	0	0
ASV48	0	3	1	4
ASV109	0	4	0	4
ASV4	1	2	0	2
ASV12	0	1	2	3
ASV41	2	0	1	1
ASV68	3	0	0	0
ASV83	2	0	1	1
ASV63	0	1	2	3
ASV52	1	0	1	1
ASV147	0	2	0	2
ASV179	0	0	2	2
ASV5	0	0	2	2
ASV92	0	0	2	2
ASV89	0	0	2	2
ASV11	0	0	2	2
ASV256	2	0	0	0
ASV102	0	0	2	2
ASV362	0	0	2	2
ASV200	0	0	2	2
ASV141	0	0	2	2
ASV633	2	0	0	0
ASV140	0	1	0	1
ASV144	0	0	1	1
ASV60	1	0	0	0
ASV47	0	0	1	1
ASV106	1	0	0	0
ASV85	0	0	1	1
ASV258	0	0	1	1
ASV441	1	0	0	0
ASV15	0	0	1	1
ASV34	1	0	0	0
ASV70	0	0	1	1
ASV79	0	0	1	1
ASV72	1	0	0	0
ASV50	0	0	1	1
ASV16	0	1	0	1
ASV73	0	0	1	1
ASV125	0	0	1	1
ASV24	0	0	1	1
ASV178	0	1	0	1
ASV25	0	1	0	1
ASV268	1	0	0	0
ASV380	0	1	0	1
ASV213	0	0	1	1
ASV45	0	0	1	1
ASV186	0	0	1	1
ASV40	1	0	0	0
ASV87	1	0	0	0
ASV107	1	0	0	0
ASV117	1	0	0	0
ASV232	1	0	0	0
ASV418	0	0	1	1
ASV67	1	0	0	0
ASV245	0	0	1	1
ASV224	1	0	0	0
ASV394	0	1	0	1
ASV168	0	0	1	1
ASV1190	0	1	0	1
ASV243	0	0	1	1
ASV181	0	0	1	1
ASV119	0	1	0	1
ASV51	0	1	0	1
ASV594	0	0	1	1
ASV208	0	0	1	1
ASV226	0	1	0	1
ASV222	1	0	0	0
ASV114	0	0	1	1
ASV103	0	1	0	1
ASV56	0	1	0	1
ASV1305	1	0	0	0
ASV187	0	0	1	1
ASV430	0	0	1	1
ASV647	0	0	1	1
ASV396	0	0	1	1
ASV220	0	1	0	1
ASV455	1	0	0	0
ASV127	0	1	0	1
ASV402	1	0	0	0
ASV228	0	0	1	1
ASV285	1	0	0	0
ASV194	1	0	0	0
ASV342	0	1	0	1
ASV1447	1	0	0	0
ASV562	1	0	0	0
ASV603	0	0	1	1
ASV776	1	0	0	0
ASV381	0	0	1	1
ASV642	0	0	1	1
ASV502	1	0	0	0
ASV1181	0	0	1	1
ASV382	0	0	1	1
ASV217	0	0	1	1
ASV1064	0	0	1	1
ASV878	1	0	0	0
ASV4671	1	0	0	0
ASV368	0	0	1	1
ASV663	0	1	0	1
ASV842	1	0	0	0
ASV2233	1	0	0	0
ASV3767	0	0	1	1
ASV383	0	0	1	1
ASV1270	0	0	1	1
ASV1034	1	0	0	0
ASV1272	0	0	1	1
ASV2925	0	0	1	1
ASV1276	0	0	1	1
ASV3077	1	0	0	0
ASV1597	1	0	0	0
ASV1070	0	0	1	1
ASV4074	0	0	1	1
ASV5172	0	0	1	1
ASV4537	0	1	0	1
ASV4494	0	0	1	1
ASV3547	0	1	0	1
ASV1980	0	0	1	1
ASV5331	1	0	0	0
ASV2156	1	0	0	0
ASV4040	0	1	0	1
ASV2908	1	0	0	0
Total abundance of reads	11440	14247	10183	24430

ASV368	1	0	0	1
ASV1257	1	0	0	1
ASV537	0	0	1	1
ASV1754	1	0	0	1
ASV1991	0	0	1	1
ASV1222	0	0	1	1
ASV2233	1	0	0	1
ASV1629	0	0	1	1
ASV605	0	0	1	1
ASV798	1	0	0	1
ASV1990	1	0	0	1
ASV1303	1	0	0	1
ASV1371	0	0	1	1
ASV1042	0	1	0	1
ASV512	0	0	1	1
ASV1319	0	0	1	1
ASV1559	0	0	1	1
ASV721	0	1	0	1
ASV959	0	0	1	1
ASV5647	0	1	0	1
ASV2934	0	0	1	1
ASV5772	1	0	0	1
ASV3364	0	0	1	1
ASV5756	0	0	1	1
ASV4200	0	0	1	1
ASV3824	0	0	1	1
ASV4999	0	1	0	1
ASV3741	0	1	0	1
ASV5418	1	0	0	1
Total number of reads	1757	2245	4958	8960

Table S4: Relative abundance data of bacterial communities from sterile plants grown in sterile 0.5X MS agar and three microboxes. The numbers are colored according to different thresholds in number of reads: green, less than 5 reads; yellow, from 5 to 49 reads; red, at least 50 reads.

	Original soils	Starting points (soil substitutes just after inoculation with AG and FO communities)	Soil at outside location without plants
ASV3	Yes	Yes	Yes
ASV8	Yes	Yes	Yes
ASV5	Yes	Yes	Yes
ASV48	Yes	Yes	Yes
ASV319	No	No	No
ASV465	No	Yes	Yes
ASV83	Yes	Yes	Yes
ASV497	Yes	Yes	Yes
ASV779	No	No	No
ASV1305	No	No	No
ASV269	Yes	Yes	Yes
ASV220	Yes	No	No
ASV276	Yes	No	No
ASV682	No	No	No
ASV419	No	No	Yes
ASV407	No	No	No
ASV342	No	No	No
ASV1209	No	No	No
ASV1432	No	Yes	No
ASV389	Yes	No	Yes
ASV898	No	No	No
ASV663	No	Yes	No
ASV1128	No	No	No
ASV378	No	Yes	Yes
ASV1344	No	No	No
ASV763	No	No	No
ASV1019	No	No	Yes
ASV1029	No	No	No
Zotu178	Yes	Yes	Yes
Zotu298	Yes	Yes	Yes
Zotu1190	No	No	No
Zotu156	Yes	Yes	Yes
Zotu250	Yes	Yes	Yes
Zotu1138	No	No	No
Zotu1104	No	No	No
Zotu1364	No	No	No
Zotu443	Yes	Yes	Yes
Zotu1198	No	No	No
Zotu901	No	No	No
Zotu1214	No	Yes	No
Zotu1777	No	Yes	Yes
Zotu1995	No	No	No
Zotu2871	No	No	No

Table S5: Detection of the ASVs responsible for the convergence of floral communities or identified as being characteristic of these communities in different soil controls.

Taxon	Phylum	Class	Order	Family	Genus	Species
ASV1827	Firmicutes	Bacilli	Bacillales	Bacillaceae	Bacillus	Bacillus
ASV665	Firmicutes	Bacilli	Bacillales	Bacillaceae	Bacillus	Bacillus sp. mixed culture X5-45
ASV671	Firmicutes	Bacilli	Bacillales	Bacillaceae	Bacillus	Bacillus sp. mixed culture X5-45
ASV807	Firmicutes	Bacilli	Bacillales	Bacillaceae	Bacillus	Bacillus sp. 3F-VII

Table S6: Taxonomic annotations from the SILVA database of the four potential seed contaminants.

								Negative controls of DNA extractions																					Negative controls included during library preparation						Mock community
	Rosettes sterile plants box 2	Rosettes sterile plants box 2	Flowers sterile plants box 2	Flowers sterile plants Box 2	Rosettes sterile plants box 3	Rosettes sterile plants box 3	Flowers sterile plants box 3	NC1	NC2	NC3	NC4	NC5	NC6	NC7	NC8	NC9	NC10	NC11	NC12	NC13	NC14	NC15	NC16.2	NC17	NC18	NC19	NC21	NC20	Empty tube 1	Empty tube 2	Empty tube 3	Empty tube 4	Empty tube 5	Empty tube 6	Zymo
**ASV3**	1	2	26	37	12	9	18	47	100	20	0	6	36	6	8	46	5	5	171	2	16	85	32	8	1	1	18	1	1	2	2	2	0	2	1
**ASV8**	1	3	20	23	9	2	4	1	3	1	0	3	0	2	2	47	1	2	40	1	0	1	0	0	2	2	1	0	0	1	0	0	1	0	2
**ASV5**	0	2	5	1	0	0	2	3	0	5	0	1	1	0	3	3	2	5	3	2	1	0	4	4	6	0	4	0	0	1	0	0	0	2	3
**ASV48**	0	1	0	1	3	0	1	0	0	0	0	0	0	0	1	0	0	0	34	0	1	0	0	0	1	1	0	0	0	0	0	0	0	0	0
**ASV319**	0	0	0	0	0	0	0	0	0	0	0	0	0	0	0	0	0	0	0	0	0	0	0	0	0	0	0	0	0	0	0	0	0	0	0
**ASV465**	0	0	0	0	0	0	0	0	0	0	0	0	0	0	0	0	0	0	0	0	0	0	0	0	0	0	0	0	0	0	0	0	0	0	0
**ASV83**	0	0	0	1	0	2	1	0	1	0	0	0	0	0	0	0	0	1	0	0	13	0	0	0	0	0	0	0	0	0	0	0	0	1	0
**ASV497**	0	0	0	0	0	0	0	0	0	1	0	0	0	0	0	0	0	0	0	0	0	0	0	0	0	0	0	0	0	0	0	0	0	0	0
**ASV779**	0	0	0	0	0	0	0	0	0	0	0	0	0	0	0	0	0	0	0	0	0	0	0	0	0	0	0	0	0	0	0	0	0	0	0
**ASV1305**	0	0	0	0	0	1	0	0	0	0	0	0	0	0	0	0	0	0	1	0	0	0	0	0	0	0	0	0	0	0	0	0	0	0	0
**ASV269**	1	0	0	1	0	0	0	0	0	0	0	0	0	0	0	0	0	0	0	0	0	0	0	0	0	0	0	0	0	0	0	0	0	0	0
**ASV220**	0	1	0	1	1	0	0	0	0	0	0	0	0	0	0	0	0	0	0	0	0	0	0	0	0	0	0	0	0	0	0	0	0	0	0
**ASV276**	0	0	0	0	0	0	0	0	0	1	0	0	0	0	0	0	0	0	0	0	0	1	0	0	0	0	0	0	0	0	0	0	0	0	0
**ASV682**	0	0	0	1	0	0	0	0	0	0	0	0	0	0	0	0	0	0	0	0	0	0	0	0	0	0	0	0	0	0	0	0	0	0	0
**ASV419**	0	0	0	2	0	0	0	0	0	0	0	0	0	0	1	0	0	0	0	0	0	0	0	0	0	0	0	0	0	0	0	0	0	0	0
**ASV407**	0	0	0	0	0	0	0	0	0	0	0	0	0	0	0	1	0	0	0	0	0	0	0	0	0	0	0	0	0	0	0	0	0	0	0
**ASV342**	0	0	0	0	1	0	0	0	0	0	0	0	0	0	0	0	0	0	0	0	0	0	0	0	0	0	0	0	0	0	0	0	0	0	0
**ASV1209**	0	0	0	0	0	0	0	0	0	0	0	0	0	0	0	0	0	0	0	0	0	0	0	0	0	0	0	0	0	0	0	0	0	0	0
**ASV1432**	0	0	0	0	0	0	0	0	0	0	0	0	0	0	0	0	0	0	0	0	0	0	0	0	0	0	0	0	0	0	0	0	0	0	0
**ASV389**	0	0	0	0	0	0	0	0	0	0	0	0	0	0	0	0	0	0	0	0	0	0	0	0	0	0	0	0	0	0	0	0	0	0	0
**ASV898**	0	0	0	0	0	0	0	0	0	0	0	0	0	0	0	0	0	0	0	0	0	0	0	0	0	0	0	0	0	0	0	0	0	0	0
**ASV663**	0	0	0	0	1	0	0	0	0	0	0	0	0	0	0	0	0	0	0	0	0	0	0	0	0	0	0	0	0	0	0	0	0	0	0
**ASV1128**	0	0	0	0	0	0	0	0	0	0	0	0	0	0	0	0	0	0	0	0	0	0	0	0	0	0	0	0	0	0	0	0	0	0	0
**ASV378**	0	0	0	0	0	0	0	0	0	1	0	0	0	0	0	0	0	0	0	0	0	0	0	0	0	0	0	0	0	0	0	0	0	0	0
**ASV1344**	0	0	0	0	0	0	0	0	0	0	0	0	0	0	0	0	0	0	0	0	0	0	0	0	0	0	0	0	0	0	0	0	0	0	0
**ASV763**	0	0	0	0	0	0	0	0	0	0	0	0	0	0	0	1	0	0	0	0	0	0	0	0	0	0	0	0	0	0	0	0	0	0	0
**ASV1019**	0	0	0	0	0	0	0	0	0	0	0	0	0	0	0	0	0	0	0	0	0	0	0	0	0	0	0	0	0	0	0	0	0	0	0
**ASV1029**	0	0	0	0	0	0	0	0	0	0	0	0	0	0	0	0	0	0	0	0	0	0	0	0	0	0	0	0	0	0	0	0	0	0	0
**ASV178**	0	0	0	0	1	0	0	0	0	0	0	0	0	0	0	0	0	0	0	0	0	0	0	0	0	0	0	0	0	0	0	0	0	0	0
**ASV298**	0	0	0	0	0	0	0	0	0	0	0	0	0	10	0	0	0	1	0	0	0	0	0	0	0	0	0	0	0	0	0	0	0	0	0
**ASV1190**	0	0	0	0	1	0	0	0	0	0	0	0	0	0	0	0	0	0	0	0	0	0	0	0	0	0	0	0	0	0	0	0	0	0	0
**ASV156**	1	0	0	0	0	0	0	147	1	21	38	42	54	0	20	0	0	0	20	20	6	9	21	8	18	80	59	91	0	0	0	0	1	1	0
**ASV250**	0	0	2	0	0	0	0	51	1	60	0	0	6	0	0	30	0	0	1	0	0	0	0	3	0	0	0	0	0	0	0	0	0	0	0
**ASV1138**	0	0	0	0	0	0	0	0	0	0	0	0	0	0	0	0	2	0	0	0	0	0	0	0	0	0	0	0	0	0	0	0	0	0	0
**ASV1104**	0	0	0	0	0	0	0	0	0	0	0	0	0	0	0	0	0	0	0	0	0	0	0	0	0	0	0	0	0	0	0	0	0	0	0
**ASV1364**	0	0	0	0	0	0	0	0	0	0	0	0	0	0	0	0	0	0	0	0	0	0	0	0	0	0	0	0	0	0	0	0	0	0	0
**ASV443**	0	0	0	0	0	0	0	0	0	0	0	0	0	0	0	0	0	0	0	0	0	0	0	0	0	0	0	0	0	0	0	0	0	0	0
**ASV1198**	0	0	0	0	0	0	0	0	0	0	1	0	0	0	0	0	0	0	0	0	0	0	0	0	0	0	0	0	0	0	0	0	0	0	0
**ASV901**	0	0	0	0	0	0	0	0	0	0	0	0	0	0	0	0	0	0	0	0	0	0	0	0	0	0	0	0	0	0	0	0	0	0	0
**ASV1214**	0	0	0	0	0	0	0	0	0	0	0	0	0	0	0	0	0	0	0	0	0	0	0	0	0	0	0	0	0	0	0	0	0	0	0
**ASV1777**	0	0	0	0	0	0	0	0	0	0	0	0	0	0	0	0	0	0	0	0	0	0	0	0	0	0	0	0	0	0	0	0	0	0	0
**ASV1995**	0	0	0	0	0	0	0	0	0	0	0	0	0	0	0	0	0	0	0	0	0	0	0	0	0	0	0	0	0	0	0	0	0	0	0
**ASV2871**	0	0	0	0	0	0	0	0	0	0	0	0	0	0	0	0	0	0	0	0	0	0	0	0	0	0	0	0	0	0	0	0	0	0	0

Table S7: Number of reads detected in the experimental and library controls for the ASVs responsible for the convergence of floral communities or being identified as being characteristic of these communities. Red labels are applied when the number of reads is greater than four.

Dataset 1 (separate file): Detailed description of the treatment of raw data, with the informatic code and statistics used. ASVs are called ZOTUs in this document, following the original naming convention generated by the “unoise” algorithm of USEARCH software.

Dataset 2 (separate file): R bioinformatic code used for the statistical analyses. In this code, ASVs are called ZOTUs, following the original naming convention generated by the “unoise” algorithm of USEARCH software.

Dataset 3 (separate file): R functions defined for the purpose of this study.

Dataset 4 (separate file): 16S rRNA sequences of different species of Burkholderiaceae. The sequences were trimmed to match the region of the amplicon used in the present analysis. Each sequence name contains the taxon name, the RefSeq assembly accession, the RefSeq sequence accession, the chromosome number, and the position occupied by the entire 16S rRNA copy.

Dataset 5 (separate file): R object containing the ASV table, metadata, and taxonomic assignments used to conduct the statistical analyses. In these datasets, ASVs are called ZOTUs, following the original naming convention generated by the “unoise” algorithm of USEARCH software.
