# Supplementary material for: Capacity of soil bacteria to reach the phyllosphere and convergence of floral communities despite soil microbiota variation
Source: Proc Natl Acad Sci U S A. 2021 Oct 7;118(41):e2100150118. doi: 10.1073/pnas.2100150118 (PMC8521660; doi:10.1073/pnas.2100150118)
Supplement: Supplementary File [file pnas.2100150118.sd01.gz › SI_2_RawDataCleaning.html]

Supplemental\_1\_RawDataCleaning.md


- Primers (Amplification, indexing, sequencing)
  - Amplification primers
  - Indexing primers
- Fastq files preparation
  - Check fastq files
  - Generation of fastq file with unique barcode for each read, and a fasta file with each barcode associated to sample id.
  - Checking the frequencies of each barcode in raw data
- Demultiplexing
- Merging forward and reverse reads
- Checking the orientation of reads
- Removal of first two bases that belong to sequencing primer
- Quality filtering
- Dereplification
- Denoising
- Production of the OTU table
- Taxonomical annotation of OTUs
- BLAST of sequences for which we did not get a match in SILVA database against ncbi database

Demultiplexing and cleaning of raw sequencing data

All file pathes indicated are for information purpose only, and might not be the ones actually used during the process presented here.

# Primers (Amplification, indexing, sequencing)

## Amplification primers

799F: AACMGGATTAGATACCCKG

1193R: ACGTCATCCCCACCTTCC

## Indexing primers

### Construct for the forward index:

AATGATACGGCGACCACCGAGATCTACACXXXTGGACTGCGACTGGCGAACMGGATTAGATACCCKG

Orange = adapter that will bind the cellflow

Red = index

Black = tail used to fix indexing primers to PCR primers

Green = 799F primer

indexes:

- B5-F1: TGA
- B5-F2: ACT
- B5-F3: GTC
- B5-F4: CAG
- B5-F5: TAC

### Construct for the reverse index:

CAAGCAGAAGACGGCATACGAGATXXXXXXXXXXXXCAGCCATTTAGTGTCACGTCATCCCCACCTTCC

Orange = adapter that will bind the cellflow

Red = index

Black = tail used to fix indexing primers to PCR products one

Green = 1193R primer

B5-1: TCCCTTGTCTCC

B5-2: ACGAGACTGATT

B5-3: ACCGGTATGTAC

B5-4: TGCATACACTGG

B5-5: TGGTCAACGATA

B5-6: ATCGCACAGTAA

B5-7: GTCGTGTAGCCT

B5-8: TACAGCGCATAC

B5-9: ATCCTTTGGTTC

B5-10: AGTCGAACGAGG

B5-11: ACCAGTGACTCA

B5-12: CCAATACGCCTG

B5-13: GCAACACCATCC

B5-14: AGTCGTGCACAT

B5-15: AGTTACGAGCTA

B5-16: TTGCGTTAGCAG

B5-17: TACGAGCCCTAA

B5-18: TGTCGCAAATAG

B5-19: ACAATAGACACC

B5-20: TCTCTACCACTC

B5-21: CGATCGAACACT

B5-22: ATTGCAAGCAAC

B5-23: AGCGCTCACATC

B5-24: TCGACCAAACAC

B5-25: TGTGTTACTCCT

B5-26: TGCACAGTCGCT

B5-27: TTCTAGAGTGCG

B5-28: ACACCTGCGATC

B5-29: ATTCCTCTCCAC

B5-30: CATCGACGAGTT

B5-31: CACCACAGAATC

B5-32: GGTCTTAGCACC

B5-33: TATCGCGCGATA

B5-34: CTCTACGAACAG

B5-35: CTCCTCCCTTAC

B5-36: CGTGTTATGTGG

B5-37: ATTAGCAGCGTA

B5-38: CAAGTTTCCGCG

B5-39: CCTTGTTCACCT

B5-40: AACCAGCAGATT

B5-41: CTAGAGCTCCCA

B5-42: CACGCAGTCTAC

B5-43: ACAAACATGGTC

B5-44: TCGAAACATGCA

B5-45: TTCCCACCCATT

B5-46: AGCAGAACATCT

B5-47: GAAACATCCCAC

B5-48: CTGTCAGTGACC

B5-49: CGGATCTAGTGT

B5-50: TTCTCCATCACA

B5-51: ATTTAGGACGAC

B5-52: GGTTTAACACGC

B5-53: AGACAGTAGGAG

B5-54: GCAGATTTCCAG

B5-55: AGATGATCAGTC

B5-56: TATCACCGGCAC

B5-57: CCAGATATAGCA

B5-58: GGTCTCCTACAG

B5-59: ACAGCTCAAACA

B5-60: ATAGCGAACTCA

B5-61: AACCGCATAAGT

B5-62: CTTGAGAAATCG

B5-63: CAGTCGTTAAGA

B5-64: CTTCCAACTCAT

B5-65: AATAGCATGTCG

B5-66: AAGTCACACACA

B5-67: CACACAAAGTCA

B5-68: GTTCCTCCATTA

B5-69: CATCAAGCATAG

B5-70: CAAGCCCTAGTA

B5-71: CCTCTGAGAGCT

B5-72: ACAAGAACCTTG

B5-73: TCATTCCACTCA

B5-74: ACCATCCAACGA

B5-75: ATGCCGGTAATA

B5-76: TCAACCCGTGAA

B5-77: TCTGTAGAGCCA

B5-78: TCGGATCTGTGA

B5-79: ACTACCTCTTCA

B5-80: CTATCCAAGTGG

B5-81: AGCCAGTCATAC

B5-82: GAGTTAGCATCA

B5-83: TAAGACTACTGG

B5-84: GTCTCCTCCCTT

B5-85: CTTAGCTACTCT

B5-86: ATCGAATCGAGT

B5-87: CGCGTCAAACTA

B5-88: GATCAACCCACA

B5-89: AGTGTCGATTCG

B5-90: TCCGAGTCACCA

B5-91: AGTCTGTCTGCG

B5-92: AGACAAGCTTCC

B5-93: GATCTAATCGAG

B5-94: TAAACGCGACTC

B5-95: TCACGAGTCACA

B5-96: TTGAACAAGCCA

### Sequencing primers

F read (B5-R1):

TGGACTGCGACTGGCGAACMGGATTAGATACCC

R read (B5-R2):

CAGCCATTTAGTGTCACGTCATCCCCACCTTCC

Indexing Primer B5-index:

GGAAGGTGGGGATGACGTGACACTAAATGGCTG

Indexing primer B5-Fx:

This primer is present into the sequencing kit used.

# Fastq files preparation

## Check fastq files

fastx\_info command from USEARCH v11.0.667\_i86linux64 to check quality of reads generated from sequencer.

```
# Linux

usearch -fastx_info /nfs/home/jmassoni/SoilContribRawAndProcessData/RawData/Undetermined_S0_L001_R1_001.fastq -output /nfs/home/jmassoni/SoilContribRawAndProcessData/fastqInfo/readsR1_info.txt

usearch -fastx_info /nfs/home/jmassoni/SoilContribRawAndProcessData/RawData/Undetermined_S0_L001_R2_001.fastq -output /nfs/home/jmassoni/SoilContribRawAndProcessData/fastqInfo/readsR2_info.txt
```

Results for reads 1:

File size 13G, 19.5M seqs, 5.9G letters and quals  
Lengths min 35, lo\_quartile 300, median 300, hi\_quartile 301, max 301  
Letter freqs G 27.0%, A 25.0%, T 24.7%, C 23.3%, N 0.000%  
0% masked (lower-case)  
ASCII\_BASE=33  
EE mean 4.1; min 0.0, lo\_quartile 0.4, median 1.3, hi\_quartile 5.8, max 37.6

Results for reads 2:

File size 13G, 19.5M seqs, 5.9G letters and quals  
Lengths min 59, lo\_quartile 300, median 300, hi\_quartile 301, max 301  
Letter freqs C 31.1%, T 26.3%, G 21.5%, A 21.2%, N 0.000%  
0% masked (lower-case)  
ASCII\_BASE=33  
EE mean 9.9; min 0.0, lo\_quartile 4.4, median 8.6, hi\_quartile 15.1, max 156.6

## Generation of fastq file with unique barcode for each read, and a fasta file with each barcode associated to sample id.

In the fastq file, we concatenated the well and plate sequence indexes to get one unique barcode per read.

The fasta file contains the barcode sequences with the associated sample id as name.

```
# Python

# A script to prepare the barcode fastq file used in USEARCH to demultiplex the library. It generates the fasta file which matchtes the sample names and the correspoding barcode sequences  
  
import sys  
from Bio import SeqIO  
import argparse  
import pandas as pd  
import os  
  
parser = argparse.ArgumentParser(description="A script to concatenate indexes (barcodes) from raw fastq files generated by an illumina sequencer. The conserved names of sequences are those of indexFile1.")  
parser.add_argument("outputRepertory",action="store",metavar="output",help="The path of the output repertory.")  
parser.add_argument("-indexFile1",action="store",metavar="indexFile1",required=False,help="The first fastq file that contains the indexes being placed on the left.")  
parser.add_argument("-indexFile2",action="store",metavar="indexFile2",required=False,help="The second fastq file that contains the indexes being placed on the right.")  
parser.add_argument("-metadataFile",action="store",metavar="metadataFile",required=False,help="The metadata file that contain the barcode sequences for each samples")  
parser.add_argument("-columnNameSpleIds",action="store",metavar="columnNameSpleIds",default="SampleID",required=False,help="The column name of the column which contains the unique ids of the samples. The default value is \"SampleID\"")  
parser.add_argument("-columnNameBarcodes",action="store",metavar="columnNameBarcodes",default="BarcodeSequence",required=False,help="The name of the column of the metadata file that contain the sequences of the barcodes. The default value is \"BarcodeSequence\".")  
parser.add_argument("-execIndexMerging",action="store_true",required=False,help="If included in the call this script concatenate the index fastq files.")  
parser.add_argument("-execFastaIndex",action="store_true",required=False,help="If included in the call this script generate a fasta file of the indexes")  
args = parser.parse_args()  
  
# Function to concatenate two fastq-index sequences (the ids of the two indexes have to match)  
def concat_index(seq1, seq2):  
    newSeq = seq1.__add__(seq2)  
    if seq1.id == seq2.id:  # Checking that indexes 1 and 2 are indeed from the same cluster  
  newSeq.id = seq1.id  # Conservation of the id of the index 1  
  newSeq.description = seq1.description  # conservation of description of the index  
  else:  
        sys.exit("error: tries to concatenate two indexes from different clusters!")  
    return (newSeq)  
  
# Generator function to concatenate sequences of two fastq-index files (the id of the two indexes have to match)  
def concat_index_files(file1, file2):  
    file1 = SeqIO.parse(file1, "fastq")  
    file2 = SeqIO.parse(file2, "fastq")  
    for seq1 in file1:  
        concSeq = concat_index(seq1, next(file2))  
        yield concSeq  
  
# Concatenation of index files  
  
if args.execIndexMerging:  
  
    concatFile = concat_index_files(args.indexFile1, args.indexFile2) # the generator object that generate the concatenated barcodes  
  
  fastqFileName = os.path.join(args.outputRepertory, "concatIndexes.fastq")  
  
    with open(fastqFileName, "w") as conFile:  
        for seq in concatFile:  
            conFile.write(seq.format("fastq"))  
  
# Generation of the fasta index file needed for USEARCH demultiplexing method  
  
if args.execFastaIndex:  
  
    metadataFile = pd.read_csv(args.metadataFile,sep="\t",header=0,index_col=None)  
  
    fastaFileName = os.path.join(args.outputRepertory, "barcodes.fasta")  
  
    with open(fastaFileName, "w") as barcFile:  
        for spl, sequence in zip(metadataFile[args.columnNameSpleIds], metadataFile[args.columnNameBarcodes]):  
            barcFile.write(">" + spl + "\n" + sequence + "\n")
```

## Checking the frequencies of each barcode in raw data

Production of a tsv file, which summarizes frequency of each barcode in data.

```
# Linux

data="/nfs/home/jmassoni/SoilContribRawAndProcessData/ProcessData/concatIndexes.fastq"
cat ${data} | grep "^@M" -A 1 | grep "^@M" -v | grep "\-\-" -v | awk '{print substr($0,0,15)}' | sort | uniq -c | sort -nr > sumDataBarcodes.txt
```

Checking that all barcodes indicated in the metadata file are present in the raw data.

```
#python

# Check that all barcodes present in the metaFile are present in the raw data  
  
import pandas as pd  
import re  
  
# Correcting the file sumDataBarcodes.txt that was generated with spaces at the begining of each lines (removal of these spaces).  
with open("Z:\SoilContribRawAndProcessData\ProcessData\sumDataBarcodesCorrected.txt", "w") as barcodeFileCorrect:  
    with open("Z:\SoilContribRawAndProcessData\ProcessData\sumDataBarcodes.txt", "r") as barcodeFile:  
        for line in barcodeFile:  
            barcodeFileCorrect.write(re.sub("^ *(?=[0-9])", "", line))  
  
barcodeFile = pd.read_csv("Z:\SoilContribRawAndProcessData\ProcessData\sumDataBarcodesCorrected.txt",sep=" ",header=None,index_col=None,encoding="UTF-8")  
  
metadataFile = pd.read_csv("C:\\Users\jmassoni\Work FolDers\Documents\Angio_Micro\SoilContributionProject\Analyses\metaDataFinal.tsv",header=0,index_col=None,sep="\t")  
  
notInData = set(metadataFile["BarcodeSequence"].tolist()) - set(barcodeFile.loc[:,1].tolist()) # OK all the barcodes present in the metadata file are also present in the raw data.
```

# Demultiplexing

To demultiplex the library, we used the -fastx\_demux command of USEARCHv11.0.667\_i86linux64.

```
#linux

usearch -fastx_demux /nfs/home/jmassoni/SoilContribRawAndProcessData/RawData/Undetermined_S0_L001_R1_001.fastq -reverse /nfs/home/jmassoni/SoilContribRawAndProcessData/RawData/Undetermined_S0_L001_R2_001.fastq -index /gram/vorholt/jmassoni/OMICS/jmassoni/SoilContribRawAndProcessData/ProcessData/concatIndexes.fastq -barcodes /gram/vorholt/jmassoni/OMICS/jmassoni/SoilContribRawAndProcessData/ProcessData/barcodes.fasta -fastqout /gram/vorholt/jmassoni/OMICS/jmassoni/SoilContribRawAndProcessData/ProcessData/Undetermined_S0_L001_R1_001.fastq -output2 /gram/vorholt/jmassoni/OMICS/jmassoni/SoilContribRawAndProcessData/ProcessData/Undetermined_S0_L001_R2_001.fastq
```

We renamed the outputs as: Demultiplexed\_S0\_L001\_R1\_001.fastq and Demultiplexed\_S0\_L001\_R2\_001.fastq.

With the following script, we tested that in the demultiplexed read 1 and read 2 fastq files the barcodes associated with reads of samples were those expected according to our metadata file.

```
#python

# A script to check that the demultiplexing procedure of USEARCH worked  
  
import argparse  
from Bio import SeqIO  
import pandas as pd  
import sys  
  
parser = argparse.ArgumentParser(description="A script to check that the barcodes of demultiplexed reads and expected ones from the metadata file match.")  
parser.add_argument("-demRdFilePath",action="store",metavar="demultiplexed read fastq file",required=True,help="The fastq file which contains the demultiplexed reads with the sample id included within the id of the cluster (format: id of the cluster;sample=id).")  
parser.add_argument("-concatIdxFastqFilePath",action="store",metavar="Barcode file",required=True,help="The fastq file which contains the barcodes used to demultiplex.")  
parser.add_argument("-metaDataPath",action="store",metavar="metadata file",required=True,help="The metadata file which contains the barcodes for each samples.")  
parser.add_argument("-columnSamples",action="store",metavar="Name of column of sample ids in metadata.",default="SampleID",required=False,help="The name of the column where the barcodes of each sample are stored in the metadata file.")  
parser.add_argument("-columnBarcodes",action="store",metavar="Name of column of barcodes in metadata.",default="BarcodeSequence",required=False,help="The name of the column where the sample ids are stored in the metadata file.")  
parser.add_argument("-numbSpleTested",action="store",default=1,type=int,metavar="Number of samples to test.",required=False,help="The number of samples to test with this script.")  
parser.add_argument("-limitNumbIdPicked",action="store",default=True,metavar="Limit the number of cluster id picked for each sample",required=False,help="The first step is to identify in the demultiplexed fastq file the ids of illumina clusters that were associated with one or more sample. In general there must be thousands of them, so you can limit the number extracted in the present script")  
parser.add_argument("-numbIdPicked",action="store",type=int,default=1,metavar="Number of cluster ids picked for each sample",required=False,help="Number of ids to pick for each sample if we do not want to collect all of them (limitNumbIdPicked=True).")  
args = parser.parse_args()  
  
metaData = pd.read_csv(args.metaDataPath,sep="\t",header=0,index_col=None) # metadata  
  
splesToTest = metaData[args.columnSamples].sample(n=args.numbSpleTested).tolist() # Random sampling to test that demultiplexing did work  
  
# A function to extract the ids of illumina cluster associated with specific samples in fastq files with the sample names indicated in id of the sequence.  
def extr_clust_id(fastqFilePath, sample, limitNumbIdPicked, nbIdToPick): # limitNumbIdPicked limits the number of sequences considered for one sample.  
  readFile = SeqIO.parse(fastqFilePath, "fastq")  # Initialistation of the iterator of the fastq file  
  listIds = [] # The list of collected ids  
  patternToFind = "sample=" + str(sample) + ";" # the pattern to find in the sequence id.  
  if limitNumbIdPicked:  
        while len(listIds) < nbIdToPick:  
            sequence = next(readFile)  
            if patternToFind in sequence.id: # Identification of sequence ids of the sample  
  listIds.append(sequence.id.split(";")[0]) # Extraction of the cluster id  
  else:  
        seq = next(readFile)  
        if patternToFind in sequence.id:  # Identification of sequence ids of the sample  
  listIds.append(sequence.id.split(";")[0])  # Extraction of the cluster id  
  return listIds  
  
# A function to extract the barcode of a cluster of illumina seq from fastq files  
def extr_barcode_clust(fastqFile, id):  
    readFile = SeqIO.parse(fastqFile, "fastq")  
    continueSearch = True  
 while continueSearch:  
        sequence = next(readFile)  
        if id in sequence.id:  
            continueSearch = False  
 return sequence  
  
for sple in splesToTest:  
    clusterId = extr_clust_id(fastqFilePath=args.demRdFilePath, sample=sple, limitNumbIdPicked=args.limitNumbIdPicked, nbIdToPick=args.numbIdPicked) # the cluster used to test demultiplexing of this sample  
  barcodeCluster = str(extr_barcode_clust(fastqFile=args.concatIdxFastqFilePath, id=clusterId[0]).seq) # the barcode associated with the cluster  
  barcodeSple = metaData.loc[metaData[args.columnSamples]==sple,args.columnBarcodes].to_string(index=False)  
    if barcodeCluster == barcodeSple:  
        print("For sample \"" + sple + "\" barcodes in metadata file and in demultiplexed reads match !")  
    else:  
        sys.exit("WARNING: barcodes do not match for " + sple + "!")
```

# Merging forward and reverse reads

```
#linux

usearch -fastq_mergepairs /gram/vorholt/jmassoni/OMICS/jmassoni/SoilContribRawAndProcessData/ProcessData/Demultiplexed_S0_L001_R1_001.fastq -reverse /gram/vorholt/jmassoni/OMICS/jmassoni/SoilContribRawAndProcessData/ProcessData/Demultiplexed_S0_L001_R2_001.fastq -fastqout /gram/vorholt/jmassoni/OMICS/jmassoni/SoilContribRawAndProcessData/ProcessData/mergedReads2.fastq -report reportMerging.txt -tabbedout tableMerging.txt -fastq_maxdiffs 20 -fastq_pctid 70 -fastq_minmergelen 250
```

Results:

Merged length distribution:  
250 Min  
375 Low quartile  
379 Median  
380 High quartile  
582 Max

Totals:  
10293303 Pairs (10.3M)  
5232146 Merged (5.2M, 50.83%)  
675859 Alignments with zero diffs (6.57%)  
3380264 Too many diffs (> 20) (32.84%)  
24 Fwd tails Q <= 2 trimmed (0.00%)  
0 Rev tails Q <= 2 trimmed (0.00%)  
24 Fwd too short (< 64) after tail trimming (0.00%)  
0 Rev too short (< 64) after tail trimming (0.00%)  
892806 No alignment found (8.67%)  
0 Alignment too short (< 16) (0.00%)  
788063 Merged too short (< 250)  
805401 Staggered pairs (7.82%) merged & trimmed  
222.13 Mean alignment length  
378.22 Mean merged length  
0.89 Mean fwd expected errors  
6.16 Mean rev expected errors  
0.24 Mean merged expected errors

# Checking the orientation of reads

Here we use a the Silva database Nr 99 (release 132) to check the orientation of our reads, and orient them in the same direction.

```
#linux

usearch -orient /gram/vorholt/jmassoni/OMICS/jmassoni/SoilContribRawAndProcessData/ProcessData/mergedReads.fastq -db /gram/vorholt/jmassoni/OMICS/jmassoni/SoilContribRawAndProcessData/SilvaDataBase/SILVA_132_SSURef_Nr99_tax_silva.fasta -fastqout /gram/vorholt/jmassoni/OMICS/jmassoni/SoilContribRawAndProcessData/ProcessData/mergedReadsOrient.fastq
```

Results: 5222849 plus (99.8%), 0 minus (0.0%), 9297 undet. (0.2%)

Because apparently all sequences are in the same direction, and for few of them the algorithm could not decide, we continued to work with the file mergeReads.fastq.

# Removal of first two bases that belong to sequencing primer

```
#linux

usearch -fastx_truncate /gram/vorholt/jmassoni/OMICS/jmassoni/SoilContribRawAndProcessData/ProcessData/mergedReads.fastq -stripleft 2 -fastqout /gram/vorholt/jmassoni/OMICS/jmassoni/SoilContribRawAndProcessData/ProcessData/mergedReadsStripped.fastq
```

# Quality filtering

We excluded any read that had more than one expected error.

```
#linux

usearch -fastq_filter /gram/vorholt/jmassoni/OMICS/jmassoni/SoilContribRawAndProcessData/ProcessData/mergedReadsStripped2.fastq -fastq_maxee 1.0 -fastqout /gram/vorholt/jmassoni/OMICS/jmassoni/SoilContribRawAndProcessData/ProcessData/mergedReadsStrippedFiltered2.fastq
```

Results:  
95.5% passed  
5232146 Reads (5.2M)  
232869 Discarded reads with expected errs > 1.00  
4999277 Filtered reads (5.0M, 95.5%)

# Dereplification

At this step, we imposed a minimum abundance of two sequences for each unique read (deletion of singletons).

```
#linux

usearch -fastx_uniques /gram/vorholt/jmassoni/OMICS/jmassoni/SoilContribRawAndProcessData/ProcessData/mergedReadsStrippedFiltered2.fastq -fastqout /gram/vorholt/jmassoni/OMICS/jmassoni/SoilContribRawAndProcessData/ProcessData/mergedReadsStrippedFilteredUniq2.fastq -fastaout /gram/vorholt/jmassoni/OMICS/jmassoni/SoilContribRawAndProcessData/ProcessData/mergedReadsStrippedFilteredUniq2.fasta -sizeout -minuniquesize 2 -strand both
```

results: 244687 uniques written, 580078 clusters size < 2 discarded (32.4%)

# Denoising

We used the denoising strategy that aims to identify OTUs at a 100% identity, after correction for potential sequencing errors and removal of chimeras.

Clusters with less than 8 reads are discarded.

```
#linux

usearch -unoise3 /gram/vorholt/jmassoni/OMICS/jmassoni/SoilContribRawAndProcessData/ProcessData/mergedReadsStrippedFilteredUniq2.fastq -zotus /gram/vorholt/jmassoni/OMICS/jmassoni/SoilContribRawAndProcessData/ProcessData/mergedReadsStrippedFilteredUniqZotus2.fasta -tabbedout /gram/vorholt/jmassoni/OMICS/jmassoni/SoilContribRawAndProcessData/ProcessData/denoising2.txt -sizeout
```

Results:  
8311 amplicons, 941402 bad (size >= 8)  
6126 good, 2185 chimeras

# Production of the OTU table

```
#linux

usearch -otutab /gram/vorholt/jmassoni/OMICS/jmassoni/SoilContribRawAndProcessData/ProcessData/mergedReadsStripped2.fastq -otus /gram/vorholt/jmassoni/OMICS/jmassoni/SoilContribRawAndProcessData/ProcessData/mergedReadsStrippedFilteredUniqZotus2.fasta -id 0.97 -strand both -otutabout /gram/vorholt/jmassoni/OMICS/jmassoni/SoilContribRawAndProcessData/ProcessData/zotutab2.txt -biomout /gram/vorholt/jmassoni/OMICS/jmassoni/SoilContribRawAndProcessData/ProcessData/zotutab2.json
```

Results: 4844372 / 5232146 mapped to OTUs (92.6%)

# Taxonomical annotation of OTUs

Finding for each sequences matches in the SILVA database at 90% identity. Only the best hits are reported. In case of ties, all of them are reported in the table.

```
#linux

usearch -usearch_global /gram/vorholt/jmassoni/OMICS/jmassoni/SoilContribRawAndProcessData/ProcessData/mergedReadsStrippedFilteredUniqZotus2.fasta -db /gram/vorholt/jmassoni/OMICS/jmassoni/SoilContribRawAndProcessData/SilvaDatabase/SILVA_132_SSURef_Nr99_tax_silva.fasta -id 0.9 -maxaccepts 20 -maxrejects 500 -strand both -top_hits_only -output_no_hits -blast6out /gram/vorholt/jmassoni/OMICS/jmassoni/SoilContribRawAndProcessData/ProcessData/90id_Zotus2.txt -threads 40
```

Results:  
99.3% matched / 40 sequences did not get any hits in the Silva database.

```
#python

# This script create a table with the sequences for which there were no matches in the database and a table with the consensus of taxonomical identifications in ties.  
  
import pandas as pd  
from pandas import read_csv  
from Bio import SeqIO  
  
rawTaxData = read_csv("Z:\SoilContribRawAndProcessData\ProcessData\\90id_Zotus2.txt", sep = "\t", header=None, index_col=None)  
  
taxWithoutMatch = rawTaxData.loc[rawTaxData[11]=="0",] # Filtering of the data for sequences without any match in the database  
  
sequences = list(SeqIO.parse("Z:\SoilContribRawAndProcessData\ProcessData\mergedReadsStrippedFilteredUniqZotus2.fasta", "fasta"))  
  
sequenceNoMatch = [seq for seq in sequences if seq.id in taxWithoutMatch[0].tolist()] # Extraction of sequences without a match  
  
with open("Z:\SoilContribRawAndProcessData\ProcessData\\90id_Zotus2_NoMatches.fasta", "w") as fastaNoMatch: # Writing of the sequences without a match in a fasta file.  
  for seq in sequenceNoMatch:  
  SeqIO.write(seq, fastaNoMatch, "fasta")  
  
taxDataFilt = rawTaxData.loc[rawTaxData[11]=="*",] # Filtering of tax data with a match in the database  
  
# A function to extract consensus of identifications when there was a tie  
def common_prefix(listStrings):  
  identif = ""  
  for i in zip(*listStrings):  
  if len(set(i))==1:  
  identif = identif + i[0]  
        else:  
  break  
 return identif  
  
# A function to exclude the ids of SILVA database at the beginning to identifications in the list of ties  
def trimTaxIdentif(listStrings):  
  newList = [" ".join(i.split(" ")[1:]) for i in listStrings]  
    return newList  
  
dictTax = {} # Initialisation of a dictionary to store the consensus  
  
for tax in taxDataFilt[0].unique():  
  listIdentif = taxDataFilt.loc[taxDataFilt[0]==tax,1].tolist() # Extraction of the ties found in SILVA for the OTU  
  listIdentifTrimmed = trimTaxIdentif(listIdentif) # Trimming of the ids from identifications  
  commonId = {tax: common_prefix(listIdentifTrimmed)}  
    dictTax.update(commonId)  
  
finalTax = pd.DataFrame.from_dict(dictTax, orient="index") # Creation of a new dataframe to store the consensus of ties  
finalTax = finalTax[0].str.split(";", expand = True) # Split identification across several columns, which correspond to different taxonomical levels  
  
finalTax.to_csv("Z:\SoilContribRawAndProcessData\ProcessData\\90id_Zotus2Consensus.tsv", header=False, index=True, sep="\t")
```

# BLAST of sequences for which we did not get a match in SILVA database against ncbi database

To check the identity of sequences for which we did not get a match in the SILVA database, we used BLAST against nt-NCBI database.

Creation of a summary from the XML files downloaded from NCBI.

```
#python

# A script to extract results of XML files generated by BLAST  
  
from bs4 import BeautifulSoup  
import os, glob, pickle  
  
listFiles = glob.glob(os.path.join("Z:\SoilContribRawAndProcessData\ProcessData", "90id_Zotu2_NoMatchesBLAST*.xml"))  
  
blastdict = {} # A dictionary to store sequence names as keys, and hit lists as values  
  
for file in listFiles: # For each file  
  with open(file, "r") as xmlFile:  
  soup = BeautifulSoup(xmlFile, "xml") # Creation of the soup  
  
  for iteration in soup.find_all("Iteration"): # Iteration across queries  
  sequence = iteration.find("Iteration_query-def").string # Extraction of the name of the sequence as in FASTA file  
  hits = [hit.string for hit in iteration.find_all("Hit_def")] # Extraction of names of all hits found for the query  
  blastdict.update({sequence: hits}) # Storage of the results in the dictionary  
  
with open("Z:\SoilContribRawAndProcessData\ProcessData\90id_Zotu2_NoMatchesBLASTSummary.txt", "w") as sumFile:  
  for key, value in blastdict.items():  
  sumFile.write(key + "\n\n")  
        for hit in value:  
  sumFile.write(hit + "\n")  
        sumFile.write("\n")
```

The lists bellow includes all sequences that got at least one Eukaryotic hit in the first 100 hits. Their exclusion are conducted in the R script for statistical analyses.

- Zotu5240
- Zotu5262
- Zotu5301
- Zotu5341
- Zotu6043
- Zotu6090
- Zotu95
- Zotu425
- Zotu521
- Zotu813
- Zotu1088
- Zotu1223
- Zotu1438
- Zotu1844
- Zotu2185
- Zotu3423
- Zotu3984
- Zotu4038
- Zotu4157
- Zotu4314
- Zotu4754
- Zotu4967
- Zotu5103
